# Pharmacogenomics of direct oral anticoagulants: a systematic review and meta-analysis

**DOI:** 10.3389/fcvm.2026.1792186

**Published:** 2026-04-14

**Authors:** Huaru Chai, Jiani Huo, Hao Chen

**Affiliations:** 1 Beijing Hospital, National Center of Gerontology, Institute of Geriatric Medicine, Chinese Academy of Medical Sciences &Peking Union Medical College, Beijing, China; 2 Cardiovascular Department, Beijing Hospital, National Center of Gerontology, Institute of Geriatric Medicine, Chinese Academy of Medical Sciences, Beijing, China

**Keywords:** *ABCB1*, *ABCG2*, bleeding risk, *CES1*, DOACs, gene polymorphism, *SLCO1B1*

## Abstract

**Background:**

Direct oral anticoagulants (DOACs) exhibit considerable individual variability in effectiveness and bleeding risk, possibly due to genetic differences. This study assessed how genetic polymorphisms impact the pharmacokinetics (PK) and outcomes of DOACs.

**Methods:**

We searched the PubMed, Embase, Web of Science, and Cochrane Library databases for pharmacogenomic studies related to DOACs up to October 29, 2025. Meta-analyses were performed using RevMan 5.4 for evaluated results with ≥3 studies.

**Results:**

Thirty-nine studies involving 13,300 patients were included, with 19 studies eligible for meta-analysis. For dabigatran, carriers of the *CES1* rs2244613 C allele was associated with both lower trough concentration (C_trough_) and reduced bleeding risk compared with AA homozygotes. *CES1* rs8192935 and *ABCB1* rs4148738 were also associated with dabigatran exposure. For rivaroxaban, the *ABCB1* rs1045642 TT genotype was consistently associated with lower dose-adjusted C_trough_ across four subgroups. Polymorphisms in *ABCB1* rs1045642 were linked to altered bleeding risk, whereas *ABCB1* (rs1128503, rs4148738, rs2032582), *ABCG2* rs2231142, *CYP3A5* rs776746, and *CYP2J2* rs890293 showed no statistically significant association with bleeding events. For apixaban, *ABCG2* rs2231142 may influence PK profiles, while *ABCB1* rs1045642 was associated with a reduced risk of bleeding. In the case of edoxaban, polymorphisms in *SLCO1B1* may affect metabolite exposure and contribute to variability in bleeding risk.

**Conclusion:**

Genetic polymorphisms in *CES1*, *ABCB1*, and *SLCO1B1* are associated with variability in the PK and bleeding risk of DOACs. However, due to the observational nature, heterogeneity, and limited sample sizes of included studies, current evidence is insufficient to support genotype-guided dosing in clinical practice. Large prospective studies are needed to validate these findings.

**Systematic Review Registration:**

PROSPERO CRD420251240030.

## 1 Introduction

Direct oral anticoagulants (DOACs), including direct factor Xa inhibitors (rivaroxaban, apixaban, edoxaban) and direct thrombin inhibitors (dabigatran) (1), have fundamentally transformed the landscape of anticoagulation therapy over the past decade. Compared with warfarin, DOACs share many advantages, including rapid onset of action, shorter half-life, fewer drug and food interactions, no requirement for routine coagulation monitoring, and lower risk of major bleeding (MB) (2, 3). As a result, DOACs have become first-line agents for stroke prevention in non-valvular atrial fibrillation (NVAF) and for the treatment and secondary prevention of venous thromboembolism (VTE) (4, 5).

The utilization of DOACs, supported by accumulating clinical evidence and guideline updates, has surged globally. In the United States, the number of DOAC users escalated from 0.20 million in 2011 to 3.50 million in 2019, with the proportion of DOAC prescriptions among oral anticoagulants rising from 7.4% to 66.8% (6). Similarly, a 2015–2019 study in China documented a rapid increase in DOAC prescriptions, with rivaroxaban surpassing warfarin for several indications (7). It is anticipated that DOAC utilization will continue to rise as the global population ages and the burden of chronic diseases grows.

Despite fixed dosing and relatively predictable pharmacokinetic (PK) and pharmacodynamic (PD) properties, considerable interindividual variability in DOAC response persists, manifesting as unpredictable bleeding or thromboembolic events. While clinical factors (e.g., age, sex, renal function, comedications) contribute to this variability, they provide an incomplete explanation (3, 8). Therefore, pharmacogenomic influences have gained increasing attention. Genes involved in the metabolism (*CES1*, *CYP450* isoforms), transport (*ABCB1*, *ABCG2*, *SLCO1B1*), and elimination of DOACs harbor polymorphisms that may modulate drug exposure and clinical outcomes (9–11).

Although numerous studies and genome-wide association studies (GWAS) have explored these relationships, findings are often inconsistent and fragmented, hampered by heterogeneity in population ethnicity, sample size, and outcome definitions. A comprehensive, quantitative synthesis—particularly one that integrates pharmacokinetic data with clinical endpoints across all major DOACs—is currently lacking (2, 12). Such a synthesis is crucial to identify robust genetic markers and assess their potential for personalized therapy.

To address this gap, we conducted this systematic review and meta-analysis. Our objectives were to: (1) synthesize the current evidence on the impact of key genetic polymorphisms on DOAC pharmacokinetics and clinical outcomes (bleeding and thrombosis); (2) perform quantitative meta-analyses where feasible to resolve inconsistencies and estimate effect sizes; (3) identify critical gaps in the literature to inform the design of future studies aimed at developing genotype-guided dosing strategies.

## 2 Materials and methods

### 2.1 Search strategy

This systematic review adhered to Systematic Reviews and Meta-Analyses (PRISMA) guidelines (13). The protocol was registered on PROSPERO under the following ID: CRD420251240030. We searched PubMed, Embase, Web of Science, and the Cochrane Library for English-language studies published from inception until October 29, 2025. The search strategy included: (dabigatran OR apixaban OR rivaroxaban OR edoxaban) AND (*CES1* OR *ABCB1* OR *ABCG2* OR *SLCO1B1* OR *CYP3A4* OR *CYP3A5* OR pharmacogenomics OR pharmacogenetics OR gene polymorphism), as shown in Supplementary Table 1.

### 2.2 Inclusion and exclusion criteria

The study selection process was independently performed by two reviewers. Any discrepancies regarding eligibility were resolved through discussion or by consulting a third reviewer. Eligibility criteria were defined according to the PICOS framework: (1) Population: patients receiving continuous therapy (≥3 days) with a DOAC (dabigatran, rivaroxaban, apixaban, or edoxaban) for approved indications (e.g., NVAF, VTE); (2) Intervention: genotyping of at least one single nucleotide polymorphism (SNP) in genes relevant to DOAC pharmacokinetics or pharmacodynamics; (3) Comparison: different genotype groups (e.g., wild-type vs. variant carriers); (4) Outcomes: PK parameters [e.g., trough concentration [C_trough_], peak concentration [C_peak_], area under the curve [AUC]]; and/or clinical endpoints (e.g., bleeding, thromboembolic events). (5) Study design: Observational studies (cohort, case-control, cross-sectional) or randomized controlled trials.

Exclusion criteria were: (1) non-English publications, or non-original research (reviews, editorials, case reports, conference abstracts, trial registry records without full text); (2) studies using a single-dose regimen or restricted to healthy volunteers; (3) duplicate publications; (4) studies including multiple DOACs without reporting outcomes separately for each drug; (5) studies involving concomitant antiplatelet therapy.

### 2.3 Data extraction and quality assessment

Data extraction was performed by one reviewer and subsequently verified by a second reviewer. Quality assessment was conducted independently by two reviewers, with any discrepancies resolved through discussion until a consensus was reached. The extracted data included: first author's surname, publication year, country, study type, patient characteristics (ethnicity, sample size, age, gender), genetic variations (gene name, rs number, minor allele frequency, Hardy-Weinberg equilibrium compliance), outcomes evaluated, and main conclusions. If an rs number was not provided in the original literature, it was retrieved using PubMed dbSNP. For PK outcomes, dose-normalized concentrations were prioritized to minimize variability arising from differences in dosing regimens. When only raw concentrations were reported, data were extracted as presented, and the absence of dose adjustment was noted. Timing of blood sample collection (trough vs. peak) was recorded and used for stratified analyses where feasible. For clarity, we categorized the studies by drug class. Results associated with each drug class will be summarized in two tables: one in the main text focusing on the association between genetic variations and outcomes evaluated (Tables 1–4); the other in the Supplementary materials detailing study design and population characteristics (Supplementary Tables 2–5).

**Table 1:** Summary of SNPs related to dabigatran in the included studies and the reported results.

Study ID	Consistent with HWE	SNP ID (Gene)	MAF	Evaluated outcomes	Conclusion
(11)	Yes	*CES1* rs2244613 *CES1* rs8192935 *ABCB1* rs4148738 *ABCB1* rs1128503	(C) = 18% (A) = 33% (G) = 45% (T) = 44%	Bleeding, ischemic events, C _trough_, C _peak_	C—C _trough_ and bleeding↓ A—C _peak_↓ G—C _peak_↑ NS
(14)	Yes	*CES1* rs2244613 *ABCB1* rs1045642 *ABCB1* rs4148738	(C) = 20.3% (T) = 50% (A) = 39.6%	Bleeding, C _trough_	CC—C _trough_↓ NS NS
(15)	Yes	*CES1* rs2244613 *CES1* rs8192935 *ABCB1* rs1045642 *ABCB1* rs4148738	(A) = 36.9% (G) = 31.3% (T) = 34.1% (G) = 41.9%	Bleeding, C _trough_, C_peak_ APTT at peak and trough, TT at trough	A—C_trough_ and minor bleeding↑ G—C_trough_ and APTT at trough↑ NS NS
(16)	NR	*UBASH3B* rs2276408 *FBN2* rs3805625	(T) = 10.1% (T) = 10.7%	Bleeding, thromboembolic and major adverse cardiac events	T—Bleeding↑ T—Bleeding↑
(17)	Yes	*CES1* rs2244613 *CES1* rs8192935 *ABCB1* rs1045642 *ABCB1* rs4148738	(A) = 40.2% (G) = 22.8% (T) = 37.9% (G) = 39.7%,	Bleeding, ischemic events, C _trough_, C _peak_	NS G—C _trough_↑ T—Bleeding↑ G—Bleeding↑
(18)	Yes	*CES1* rs2244613 *ABCB1* rs4148738	(C) = 21.53% (G) = 42.65%	Major Bleeding, ischemic events, C _trough_	C—C _trough_↓ G—Major Bleeding↑
(19)	Yes	*ABCB1* rs1045642 *ABCB1* rs4148738	(T) = 43.27% (A) = 41.35%	Bleeding	NS NS
(10)	NR	*CES1* rs2244613 *CES1* rs8192935 *ABCB1* rs1045642 *ABCB1* rs4148738 *ABCB1* rs1128503 *ABCB1* rs2032582	(C) = 17.8% (A) = 28.5% (T) = 59.4% (A) = 50.6% (T) = 47.6% (A + T) = 51.3%	Bleeding events, thromboembolic events	NS NS NS NS NS NS
(20)	Yes	*CES1* rs2244613 *ABCB1* rs1045642 *ABCB1* rs1128503 *ABCB1* rs2032582	NR	Bleeding events, thromboembolic events	C—Bleeding↓ NS NS NS
(22)	Yes	*CES1* rs2244613 *CES1* rs8192935 *ABCB1* rs4148738	(C) = 22.3% (A) = 31.5% (A) = 47.3%	C _trough_, C _peak_	NS A—C _trough_↓ NS
(24)	Yes	*CES1* rs2244613 *ABCB1* rs1045642 *ABCB1* rs4148738	(A) = 27.5% (T) = 50.8%. (A) = 45.8%	C _trough_, C _peak_	NS TT—C _peak_↑ NS
(25)	slight departure	*CES1* rs2244613 *CES1* rs8192935 *CES1* rs71647871 *ABCB1* rs1045642 *ABCB1* rs4148738 *ABCB1* rs1128503 *ABCB1* rs2032582	(C) = 57.1% (A) = 61.2% (A) = 0% (T) = 38.3% (G) = 40.4% (T) = 62.6% (A) = 56.6%	C _min_ and C _max_, APTT and TT at a minimal level	C—C min↓ A—C min↓ NS T—APTT at min level↑ A—APTT at min level↑ NS NS
(26)	slight departure	*CES1* rs2244613 *CES1* rs8192935 *ABCB1* rs4148738	(A) = 77.5% (G) = 67.5% (A) = 47.5%	C _trough_, C _peak_, dTT_trough_, dTT _peak_	NS NS GA—dTT_trough_, C _trough_↓

HWE, Hardy–Weinberg equilibrium; MAF, minor allele frequency; C _trough_ or C _min_, plasma trough concentration; C _peak_ or C _max_, plasma peak concentration; NS, no significant; NR, no reported; APTT, activated partial thromboplastin time; TT, thrombin time; dTT, diluted thrombin time.

**Table 2:** Summary of SNPs related to rivaroxaban in the included studies and the reported results.

Study ID	Consistent with HWE	SNP ID (Gene)	MAF	Evaluated outcomes	Conclusion
(27)	Yes	*ABCB1* rs1045642 *ABCB1* rs4148738 *ABCB1* rs1128503	(T) = 36.8% (G) = 40.6% (C) = 37.7%	Bleeding events, C _trough_	NS NS CC—C _trough_↓
(28)	Yes	*ABCB1* rs1045642 *ABCB1* rs4148738	(C) = 42.6% (A) = 46.5%	CRNMB, C _trough_, C _trough_/D, PT	TT—CRNMB↑ AA—CRNMB↑
(30)	Yes	*ABCB1* rs1045642 *ABCB1* rs4148738 *ABCB1* rs2032582 *CYP3A5* rs776746 *CYP2J2* rs890293 *CYP2C19* rs4244285 *CYP2C19* rs12248560	(T) = 48.6% (A) = 41.7% (G) = 32.4% (A) = 21.8% (T) = 2.3% (A) = 29.6% (T) = 1.9%	Bleeding, C_max_/D, C_min_/D	NS NS G—Bleeding, C_max_/D↑ NS NS NS NS
(31)	Yes (P>0.0001)	*ABCA6* rs7212506 *AKR7A3* rs1738023 *AKR7A3* rs1738025	NR	Bleeding	TT—Bleeding↑ CC—Bleeding↑ CC—Bleeding↑
(32)	Yes	*ABCB1* rs1045642 *ABCB1* rs4148738 *ABCB1* rs1128503 *ABCB1* rs4728709 *CYP3A4* rs2242480 *CYP3A4* rs4646437 *CYP3A5* rs776746 *ABCG2* rs2231142 *ABCG2* rs2231137	(T) = 42.1% (G) = 42.6% (C) = 36.8% (A) = 14.8% (T) = 32.5% (A) = 18.7% (A) = 30.7% (A) = 30.2% (T) = 31.3%	Bleeding, C _trough_/D	NS AA—C _trough_/D↓ NS A—C _trough_/D↓ NS NS NS NS NS
(33)	Yes	*SUSD3* rs76292544 *NCMAP* rs4553122 *PRF1* rs885821 *PRKAG2* rs12703159 *PRKAG2* rs13224758 *POU2F3* rs2298579	(T) = 13.4% (T) = 38.6% (A) = 10.4% (T) = 13% (A) = 12.8% (T) = 37.4%	Bleeding, anti-F Xa level	T—Bleeding↑ C—anti-F Xa level↑ G—anti-F Xa level↑ C—anti-F Xa level↑ G—anti-F Xa level↑ T—anti-F Xa level↑
(34)	Yes	*ABCB1* rs1045642 *ABCB1* rs4148738 *ABCB1* rs1128503 *ABCB1* rs2032582 *CYP3A5* rs776746	(C) = 41.7% (A) = 35.6% (C) = 35.6% (G) = 35.6% (A) = 22.7%	Bleeding, C _max,_ C _ss_ PT, INR, APTT	C—C _max_, C _ss_ ↓ A—C _max_, C _ss_ ↓ C—C _max_, C _ss_ ↓ G—C _max_, C _ss_ ↓ G—C _max_, C _ss_ ↓
(35)	Yes	*CYP3A4* rs2242480 *CYP3A4* rs2246709 *CYP3A4* rs3735451 *CYP3A4* rs4646440 *CYP3A5* rs776746	(T) = 23.9% (G) = 39.1% (C) = 26.1% (A) = 21.8% (A) = 25.2%	Bleeding, C _trough_, APTT, PT	T—C _trough_, PT↑ GG—C _trough_, PT↑,G—Bleeding↑ CC—C _trough_, PT↑, C—Bleeding↑ NS A—C _trough_, PT↑
(36)	Yes	*ABCB1* rs1045642 *ABCB1* rs4148738 *ABCB1* rs1128503 *ABCB1* rs2032582 *ABCG2* rs2231142 *CYP3A4* rs2740574 *CYP3A4* rs35599367 *CYP3A5* rs776746 *CYP2J2* rs11572325 *CYP2J2* rs890293	(C) = 47.3% (A) = 48.4% (T) = 44.5% (T + A) = 45.2% (A) = 9.1% (G) = 1.8% (T) = 3.0% (A) = 6.6% (T) = 11.8% (T) = 6.8%	Bleeding	NS NS NS NS NS NS NS NS NS NS
(37)	Yes	*ABCB1* rs1045642 *ABCB1* rs4148738 *ABCB1* rs1128503 *ABCB1* rs2032582 *ABCG2* rs2231142 *CYP3A4* rs35599367 *CYP3A5* rs776746 *CYP2J2* rs890293 *SUSD3* rs76292544 *AKR7A3* rs1738023 *AKR7A3* rs1738025 *ABCA6* rs7212506	(T) = 36.1% (G) = 37.8% (C) = 37.1% (T + A) = 52.7% (A) = 31.3% (T) = 0.2% (A) = 30.3% (T) = 4.2% (T) = 14.4% (T) = 21.9% (C) = 21.9% (C) = 15%	Bleeding, C _trough_, C _peak_	NS NS NS NS NS NS NS NS TT—Bleeding↑ NS NS NS
(21)	Yes	*ABCB1* rs1045642 *ABCB1* rs4148738 *ABCB1* rs1128503 *ABCB1* rs2032582	(T) = 36.1% (A) = 40.1% (C) = 37.1% (T + A) = 45.3%	Bleeding, thromboembolic events	TT—Thromboembolic events↑ GG—Bleeding↑ NS NS
(10)	Yes	*ABCB1* rs1045642 *ABCB1* rs1128503 *ABCB1* rs2032582	(T) = 58.4% (T) = 47.1% (T + A) = 50.7%	Bleeding events, thromboembolic events	T—Thromboembolic events↓ NS NS
(38)	Yes	*ABCB1* rs1045642 *ABCB1* rs4148738 *ABCB1* rs1128503 *ABCB1* rs2032582 *ABCG2* rs2231142 *CYP3A4* rs35599367 *CYP3A5* rs776746 *CYP2J2* rs890293	NR	Bleeding	NS A—Bleeding↓ NS NS NS NS G—Bleeding↓ NS
(20)	Yes	*ABCB1* rs1045642 *ABCB1* rs1128503 *ABCB1* rs2032582 *ABCG2* rs2231142 *CYP3A4* rs2242480 *CYP3A5* rs776746 *CYP2J2* rs890293	NR	Bleeding events, thromboembolic events	T—Thromboembolic events↓ NS NS NS NS NS NS
(39)	Yes	*ABCB1* rs1045642 *ABCB1* rs1128503 *ABCB1* rs2032582	(T) = 50.4% (T) = 42.7% (G) = 45.4%	AUC_0–6h_	NS NS NS
(40)	Yes^a^	*ABCB1* rs1045642 *ABCB1* rs4148738 *CYP3A4* rs35599367 C*YP3A5* rs776746	(T) = 54.5% (A) = 46.8% (T) = 3.2% (A) = 6.4%	C_max, ss_	NS NS NS NS
(41)	Yes	*ABCB1* rs1045642 *ABCB1* rs1128503 *ABCB1* rs2032582 *ABCG2* rs2231142 *CYP3A5* rs776746 *CYP2J2* rs890293	(T) = 35.5% (C) = 45.3% (G) = 47.1% (A) = 26.2% (A) = 26.7% (T) = 4.7%	C _trough_/D	NS NS NS NS NS NS
(29)	Yes	*ABCB1* rs1045642 *ABCB1* rs4148738 *CYP3A5* rs776746 *CYP3A4* rs35599367	(C) = 42.4% (G) = 46.5% (A) = 5.8% (T) = 4.1%	C _trough_, PT	NS GA—C _trough_↑ NS NS

HWE, Hardy–Weinberg equilibrium; MAF, minor allele frequency; CRNMB, clinical relevant non-major bleeding; C _trough_ or C _min_, plasma trough concentration; C _peak_ or C _max_, plasma peak concentration; NS, no significant; NR, no reported; APTT, activated partial thromboplastin time; TT, thrombin time; PT, prothrombin time.

^a^ Except for CYP3A4 rs35599367 and CYP3A5 rs776746—lack of homozygotes cannot be evaluated.

**Table 3:** Summary of SNPs related to apixaban in the included studies and the reported results.

Study ID	Consistent with HWE	SNP ID (Gene)	MAF	Evaluated outcomes	Conclusion
(42)	NR	*ABCB1* rs1045642 *ABCB1* rs1128503 *ABCB1* rs2032582 *ABCG2* rs2231142 *ABCG2* rs2231137 *CYP3A4* rs35599367 *CYP3A4* rs2740574 *CYP3A5* rs776746 *SULT1A1* rs1042028	(C) = 46.7% (T) = 46.0% (T + A) = 49.2% (A) = 11.8% (T) = 6.8% (T) = 3.7% (G) = 4.7% (A) = 9.4% (T) = 31.9%	Bleeding events, thromboembolic events, AUC _ss_, C _max, ss_, C _min, ss_	NS NS NS T—AUC _ss_, C _max, ss_, C _min, ss_↑ NS NS NS NS NS
(43)	slight departure	*ABCB1* rs1045642 *ABCB1* rs4148738 *CYP3A4* rs35599367 *CYP3A5* rs776746	(C) = 49% (G) = 44% (T) = 9.5% (A) = 3%	Bleeding, C _trough_	NS NS NS NS
(44)	Yes	*ABCB1* rs1045642 *ABCB1* rs1128503 *ABCB1* rs2032582 *CYP3A4* rs35599367 *CYP3A5* rs776746	(T) = 52.3% (T) = 42.1% (T) = 42.6% (T) = 2.7% (G) = 92.3%	Bleeding, C _min, ss_	C—Bleeding↑ NS NS NS NS
(19)	Yes	*ABCB1* rs1045642 *ABCB1* rs4148738	(T) = 49.56% (A) = 45.18%	Bleeding	NS NS
(10)	Yes	*ABCB1* rs1045642 *ABCB1* rs4148738 *ABCB1* rs1128503 *ABCB1* rs2032582 *ABCG2* rs2231142 *CYP3A5* rs776746	(T) = 57.7% (A) = 49.6% (T) = 48.4% (T + A) = 50.2% (A) = 7.4% (G) = 92.7%	Bleeding events, thromboembolic events	NS A—Bleeding events↓ NS NS NS NS
(38)	Yes	*ABCB1* rs1045642 *ABCB1* rs4148738 *ABCB1* rs1128503 *ABCB1* rs2032582 *ABCG2* rs2231142 *CYP3A4* rs35599367 *CYP3A5* rs776746 *CYP2J2* rs890293	NR	Bleeding	NS NS NS NS NS NS G—Bleeding↑ NS
(23)	Yes	*ABCB1* rs4148738	(A) = 44.9%	C _trough_, C _peak_	AA—C _peak_↓
(45)	Yes	*ABCB1* rs1045642 *ABCB1* rs1128503 *ABCB1* rs2032582 *ABCG2* rs2231142 *CYP3A5* rs776746	(T) = 40.7% (T) = 45.1% (T + A) = 55.6% (A) = 31.5% (G) = 76.5%	CL/F	NS NS NS C—CL/F↑ T—CL/F↑
(46)	Yes	*ABCB1* rs1045642 *ABCG2* rs2231142 *ABCG2* rs2231137 *CYP3A4* rs35599367 *CYP3A5* rs776746	(T) = 52.5% (A) = 11% (T) = 3.5% (T) = 5.0% (G) = 92.7%	C _trough,_ C _peak_	NS A—C _trough_, C _peak_↑ NS NS NS
(47)	Yes	*ABCB1* rs1045642 *ABCB1* rs4148738	(T) = 48.11% (A) = 46.22%	C _trough_, C _peak_	NS NS
(39)	Yes	*ABCB1* rs1045642 *ABCB1* rs1128503 *ABCB1* rs2032582	(T) = 41.6% (T) = 45.1% (T) = 46.3%	AUC_0–6h_	NS NS NS

HWE, Hardy–Weinberg equilibrium; MAF, minor allele frequency; CRNMB, clinical relevant non-major bleeding; C _trough_ or C _min_, plasma trough concentration; C _peak_ or C _max_, plasma peak concentration; NS, no significant; NR, no reported; NVAF, nonvalvular atrial fibrillation; AF, atrial fibrillation; AUC, area under the plasma concentration–time curve; CL/F, oral clearance.

**Table 4:** Summary of SNPs related to edoxaban in the included studies and the reported results.

Study ID	Consistent with HWE	SNP ID (Gene)	MAF	Evaluated outcomes	Conclusion
(9)	NR	*ABCB1* rs3842 *SLCO1B1* rs4149056 *SLCO1B1* rs4149057 *SLCO1B1* rs11045879 *SLCO1B1* rs12317268 *SLCO1B1* rs4149081 *SLCO1B1* rs999278 *SLCO1B1* rs2306283 *SLCO1B1* rs10841753 *SLCO1B1* rs2417957 *SLCO1B1* rs4149042	(C) = 32% (C) = 14% (C) = 27% (C) = 40% (G) = 38% (A) = 39% (A) = 27% (G) = 72% (C) = 31% (T) = 31% (C) = 42%	Bleeding	C—Bleeding↑ C—Bleeding↑ C—Bleeding↑ NS NS NS NS G—Bleeding↓ NS NS NS
(20)	Yes	*CES1* rs2244613 *ABCB1* rs1045642 *ABCB1* rs1128503 *ABCB1* rs2032582 *CYP3A4* rs2242480 *CYP3A5* rs776746 *SLCO1B1* rs4149056	NR	Bleeding, thromboembolic events	NS NS NS NS NS NS NS
(48)	Yes	*CES1* rs2244613 *CES1* rs8192935 *ABCB1* rs1045642 *ABCB1* rs1128503 *ABCB1* rs2032582 *CYP3A5* rs776746 *SLCO1B1* rs4149056 *SLCO1B1* rs2306283	(A) = 42.8% (G) = 24.7% (T) = 38.8% (T) = 62.8% (T + A) = 59.9% (G) = 78.6% (C) = 14.8% (G) = 63.5%	C_Edo_/D, C_M-4_/D, M-4 Ratio	NS NS NS NS NS NS C—C_M-4/D_↑ NS
(49)	Yes	*ABCB1* rs1045642 *ABCB1* rs1128503 *ABCB1* rs2032582 *CYP3A5* rs776746	(T) = 49.56% (T) = 35.9% (T + A) = 60.3% (G) = 84.0%	CL/F	NS NS NS NS

During the course of this study, we identified notable inconsistencies in SNP nomenclature and allele annotation across the published literature. For example, *ABCB1* rs1045642 has been variously reported as A>G or C>T, discrepancies that often arise from differences in strand orientation, reference genome choice, and population-specific allele frequency distributions. To ensure cross-study comparability, we systematically standardized the allele orientation for all included SNPs. For well-characterized variants, we retained their conventional designations, with allele representations aligned with those in published pharmacogenetic studies and the ClinPGX database (e.g., *ABCB1* rs1045642 is referred to as 3435C > T). For all other SNPs, we utilized the dbSNP database as the authoritative reference and uniformly converted the nomenclature to genomic coordinates based on the forward strand of the GRCh38 reference genome, following the recommendations of the Human Genome Variation Society (HGVS) (e.g., *CES1* rs8192935 is denoted as NC_000016.10: g.55827882A > G). Moreover, the effect allele was uniformly defined, as detailed in Supplementary Table 6.

For quality assessment, cohort and case-control studies used the Newcastle-Ottawa Scale (NOS), with scores ≥7 indicating high quality; 5–7 for moderate quality; <5 for low quality. Cross-sectional studies followed the Agency for Healthcare Research and Quality (AHRQ) standards with scores ≥8 as high quality; 4–7 as moderate quality; <4 as low quality.

### 2.4 Statistical analysis

Given the distinct metabolic and transporter pathways of the four DOACs, separate meta-analyses were conducted for each drug. For a given SNP, studies on the same drug were pooled only when at least three studies were available for the same endpoint. Moreover, all analyses were evaluated under four genetic models: homozygous, heterozygous, dominant, and recessive. Statistical analyses were conducted using RevMan 5.4. For dichotomous outcomes, including clinical events such as bleeding and thrombosis, we expressed effect sizes as odds ratios (ORs) with 95% confidence intervals (CIs). For continuous variables, such as C_trough_ and C_peak_, mean differences (MDs) along with their 95% CIs were used. Unadjusted and adjusted estimates were pooled separately. When both were available, the type reported by the majority of studies was used in the primary analysis.

Heterogeneity among the included studies was evaluated using Cochran's *Q* test and the *I*^2^ statistic. Significant heterogeneity was defined as *P* < 0.1 or *I*^2^ > 50%, in which case a random-effects model was applied; otherwise, a fixed-effects model was used. To investigate potential sources of heterogeneity in analyses with significant heterogeneity, we performed prespecified subgroup analyses according to study design, ethnicity, and clinical indication. Subgroup analyses were carried out only when at least three studies were available within a given subgroup. In addition, sensitivity analyses were conducted by sequentially excluding each individual study to assess its impact on the pooled effect size and on the observed heterogeneity. To ensure statistical robustness, publication bias was assessed using funnel plots and Egger's test only for meta-analyses that included at least 10 studies (50, 51). Given the observational nature of the included studies and the meta-analysis being exploratory, a GRADE assessment was not performed.

## 3 Result

**Figure 1:**
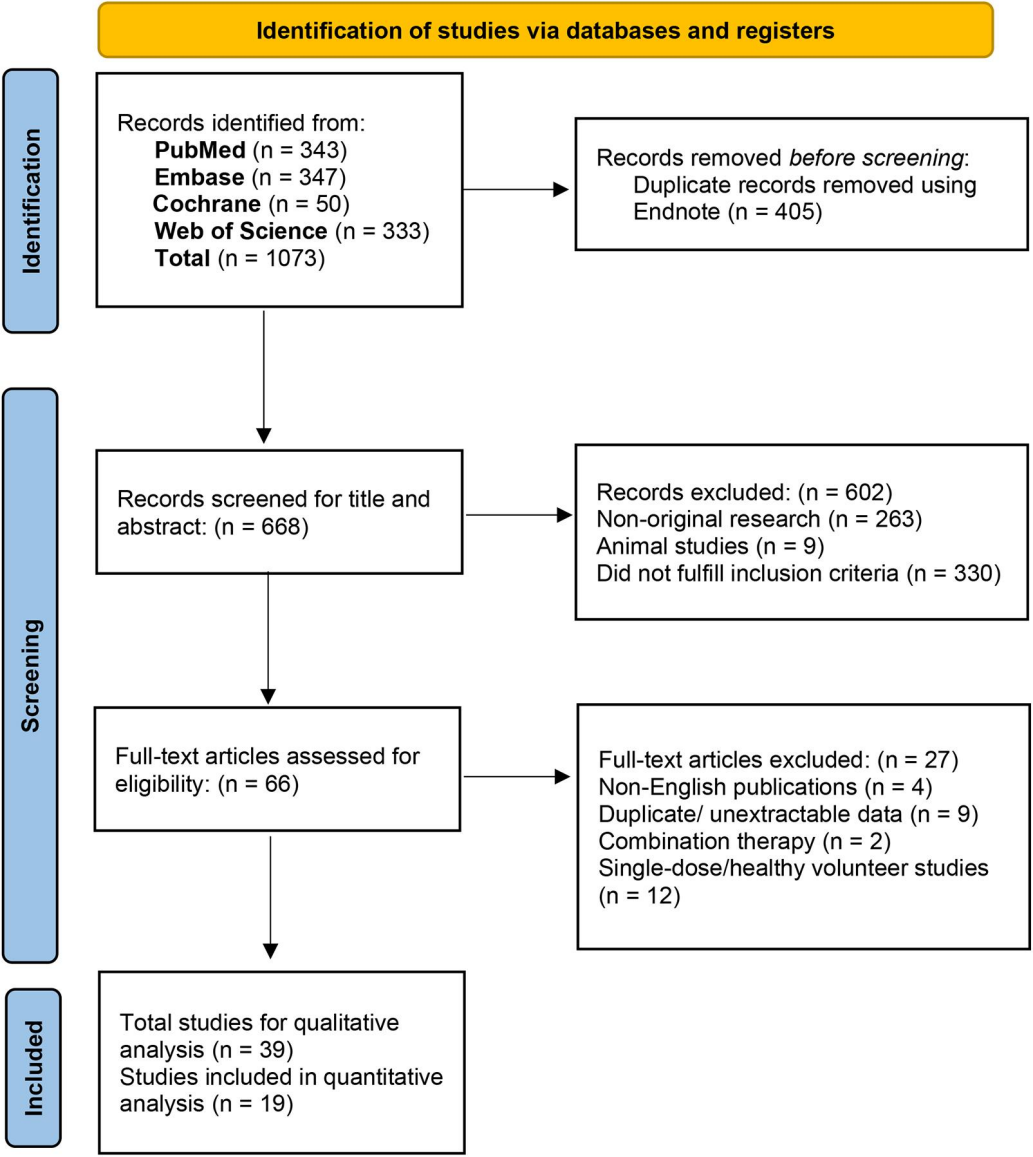
PRISMA 2020 flow diagram of study selection.

A total of 1,073 records were identified through database searching. After removing 405 duplicates, 668 records were screened by title and abstract, of which 602 were excluded (263 non-original research, 9 animal studies, 330 did not meet inclusion criteria). Full-text assessment of 66 articles led to the exclusion of 27 studies (4 non-English, 9 with duplicate or unextractable data, 2 with concomitant antiplatelet therapy, 12 single-dose or healthy volunteer studies). Ultimately, 39 studies were included in the systematic review, with 19 eligible for meta-analysis. The remaining 20 studies could not be included in the quantitative synthesis due to inadequate data or non-extractable information (e.g., lack of effect estimates, data presented in non-combinable formats). The study selection process is illustrated in Figure 1.

A total of 39 studies involving 13,300 patients were included in this review. The sample sizes ranged from 45 to 1,694, with mean patient ages ranging from 58.9 to 87.5 years. Among the 37 studies that reported gender data, males were more prevalent than females in 29 studies. The existing studies exhibit a distinct geographical distribution, with the majority conducted in Europe (*n* = 17) and Asia (*n* = 19), followed by three studies in North America. Ethnically, the study populations primarily consisted of Caucasians (*n* = 20) and Asians (*n* = 19), among which 12 studies focused specifically on Chinese populations. In terms of clinical indications, most research concentrated on patients with AF (*n* = 24), including one study that also involved patients with concurrent stage 3 chronic kidney disease. The remaining studies addressed other indications, including DOAC users (*n* = 10), VTE (*n* = 1), AF or VTE (*n* = 1), ischemic stroke (*n* = 1), and patients after joint replacement surgery (*n* = 2).

Regarding study design, the review encompassed 2 cross-sectional studies, 2 case-control studies, and 35 cohort studies, of which 27 were prospective. Most studies focused on a single oral anticoagulant (*n* = 34), while the remaining 5 studies involved two or more DOACs. Specifically, there were 13, 18, 11, and 4 studies that investigated the impact of genetic polymorphisms on dabigatran, rivaroxaban, apixaban, and edoxaban, respectively. All included studies were rated as medium to high quality, with detailed quality assessment results provided in Supplementary Tables 7, 8.

### 3.1 Dabigatran

#### 3.1.1 Pharmacokinetics and pharmacodynamics

Dabigatran etexilate, a prodrug with approximately 6.5% oral bioavailability, is rapidly absorbed and achieves C_peak_ within 1.5–3 h (52). Dabigatran etexilate undergoes near-complete hydrolysis to dabigatran via carboxylesterases, with hepatic CES1 as the primary contributor and intestinal CES2 playing a minor role (53). Dabigatran exhibits 35% plasma protein binding and is predominantly eliminated renally, with about 80% excreted unchanged in the urine. Minor hepatic metabolism occurs via glucuronidation enzymes UGT2B15, UGT1A9, and UGT2B7, yielding four pharmacologically active glucuronides (54–56). The half-life is 14–17 h in healthy patients (57). A study analyzing dabigatran trough levels in atrial fibrillation (AF) patients with chronic kidney disease (CKD) revealed an inverse correlation between renal function and C_trough_, underscoring the necessity for dose adjustment based on renal function (58). Additionally, DABE (but not active dabigatran) is a substrate for P-glycoprotein (P-gp), encoded by the ATP-binding cassette subfamily B member 1(*ABCB1*) gene, indicating that transporter-mediated interactions are confined to the absorption phase. Concomitant use of P-gp inhibitors with dabigatran has been associated with bleeding events (59). Therefore, when prescribing dabigatran with P-gp inducers (e.g., rifampicin, carbamazepine, and dexamethasone) and their inhibitors (e.g., verapamil, nicardipine, and amiodarone), drug interaction must be considered to alleviate adverse drug reactions (60).

#### 3.1.2 Pharmacogenetics of dabigatran

##### 3.1.2.1 CES1

Among more than 2,000 known polymorphisms in *CES1* (4, 61), rs2244613 and rs8192935 have been the most extensively studied.

###### 3.1.2.1.1 CES1 rs2244613

**Figure 2:**
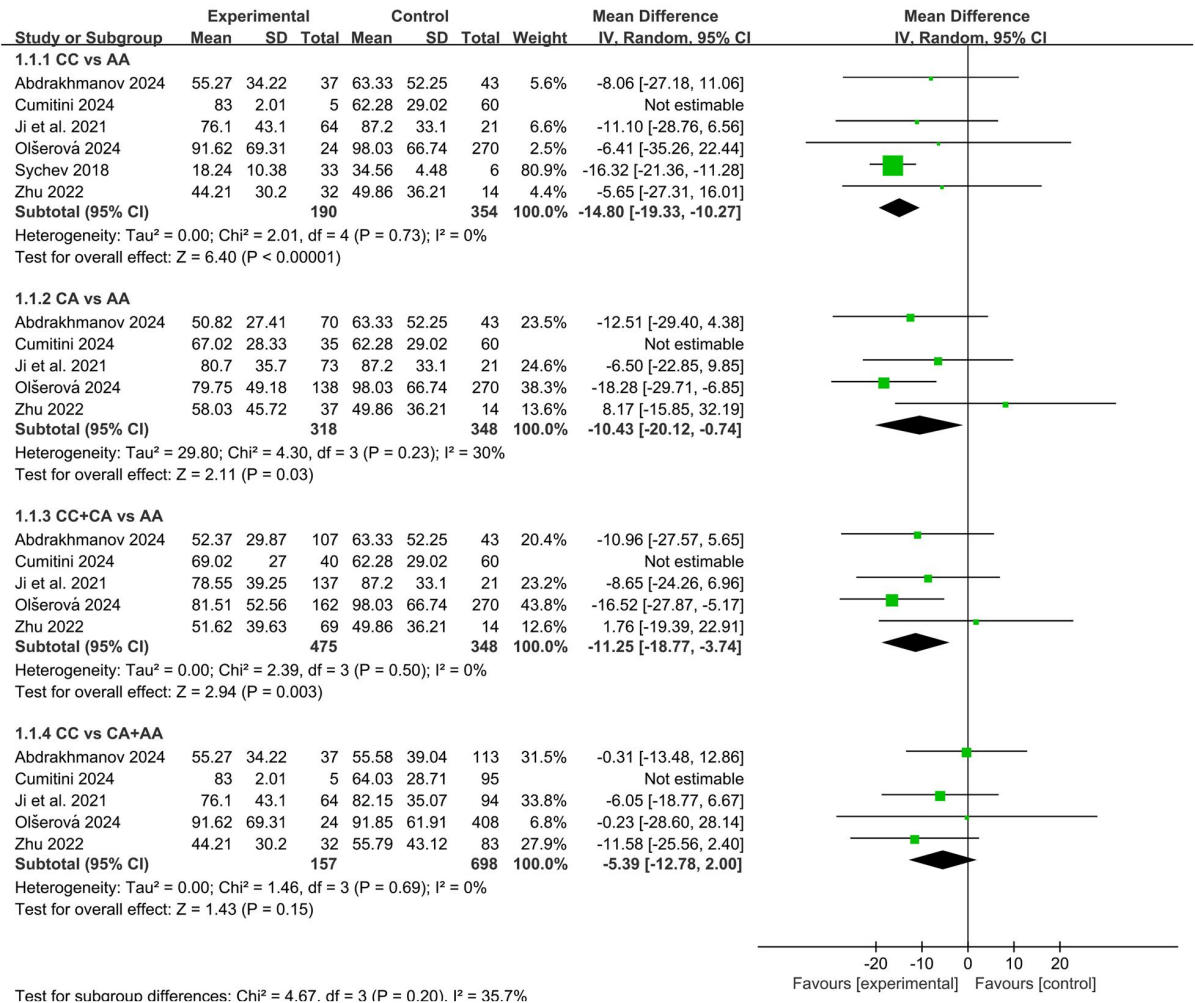
Forest plots illustrating the association between *CES1* rs2244613 and C_trough_ of dabigatran.

A total of nine studies have examined the association between *CES1* rs2244613 and C_trough_ of dabigatran (11, 14, 15, 17, 18, 22, 24–26), five of which reported significant findings (11, 14, 15, 18, 25). Six studies were eligible and included in the meta-analysis. Initial analysis revealed no significant effect of *CES1* rs2244613 on C_trough_ in any subgroup, with considerable heterogeneity observed across subgroups (I^2^ ranging from 57% to 92%). Sensitivity analysis after excluding Cumitini et al. (26) substantially reduced heterogeneity (I^2^ ranging from 0% to 30%). The pooled results demonstrated that, compared with the AA genotype, carriers of the CC genotype, CA genotype, and C allele exhibited lower C_trough_, with reductions of 14.80 ng/mL [95% CI (10.27, 19.33), *P* < 0.0001], 10.43 ng/mL [95% CI (0.74, 20.12), *P* = 0.03], and 11.25 ng/mL [95% CI (3.74, 18.77), *P* = 0.003], respectively, as shown in Figure 2.

For C_peak_, seven studies were identified (11, 15, 17, 22, 24–26), none of which reported a significant association. Three of these were included in a meta-analysis, which found no significant effect of *CES1* rs2244613 on C_peak_, as shown in Supplementary Figure 1.

**Figure 3:**
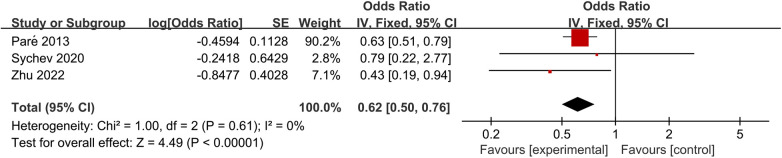
Forest plots illustrating the association between *CES1* rs2244613 and bleeding rate of dabigatran. C carriers (CC + CA) versus AA carriers.

Furthermore, seven studies explored the relationship between this SNP and bleeding risk (10, 11, 14, 15, 17, 18, 20), with three indicating significance (11, 15, 20). A meta-analysis incorporating three studies revealed that C allele carriers had a lower bleeding risk compared to AA carriers [OR = 0.62, 95% CI (0.50, 0.76), *P* < 0.00001], as shown in Figure 3.

###### 3.1.2.1.2 CES1 rs8192935

Six studies have investigated the association between *CES1* rs8192935 and the C_trough_ and C_peak_ of dabigatran (11, 15, 17, 22, 25, 26). Among them, four studies indicated that this SNP significantly affects C_trough_ (15, 17, 22, 25), while one study reported a significant effect on C_peak_ (11). Three studies were included in the meta-analysis.

**Figure 4:**
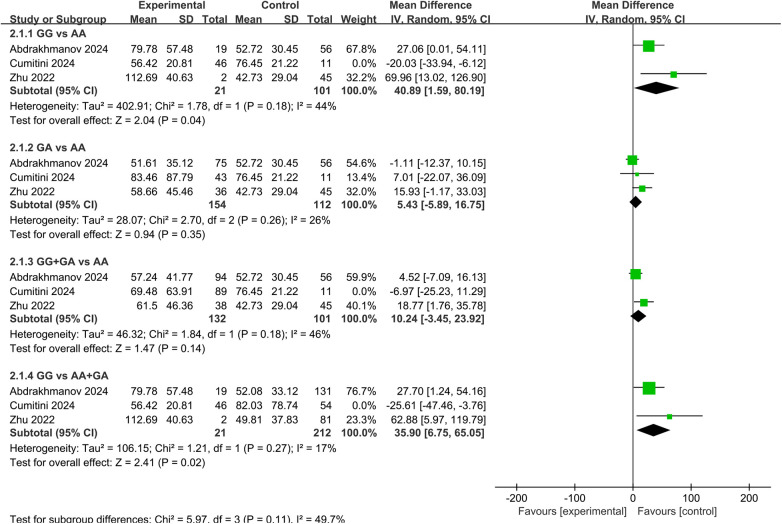
Forest plots illustrating the association between *CES1* rs8192935 and C_trough_ of dabigatran.

For C_trough_, initial pooled results suggested no significant influence of this SNP across four genetic model subgroups. However, substantial heterogeneity was observed in the homozygous, dominant, and recessive model subgroups (*I*^2^ = 88%, *P* = 0.0003; *I*^2^ = 52%, *P* = 0.13; *I*^2^ = 86%, *P* = 0.0008). After excluding Cumitini et al. (26), heterogeneity was partially resolved (*I*^2^ = 44%, *P* = 0.18; *I*^2^ = 46%, *P* = 0.18; *I*^2^ = 17%, *P* = 0.27). Further analysis revealed that GG genotype carriers had significantly higher C_trough_ compared to AA genotype carriers [MD = 40.89, 95% CI (1.59, 80.19), *P* = 0.04] and A allele carriers [MD = 35.90, 95% CI (6.75, 65.05), *P* = 0.02], as shown in Figure 4.

**Figure 5:**
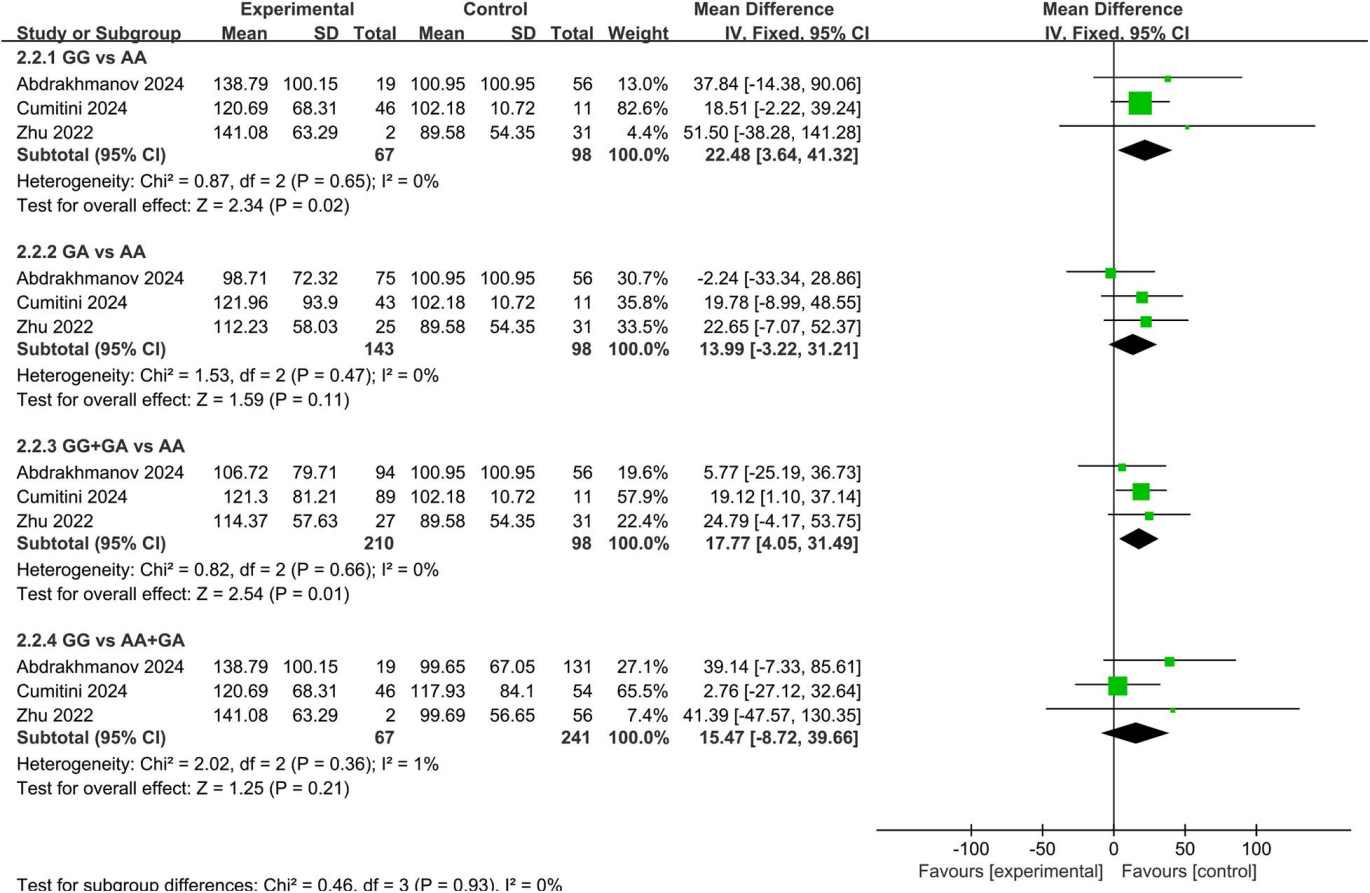
Forest plots illustrating the association between *CES1* rs8192935 and C_peak_ of dabigatran.

Regarding C_peak_, both GG genotype carriers [MD = 22.48, 95% CI (3.64, 41.32), *P* = 0.02] and G allele carriers [MD = 17.77, 95% CI (4.05, 31.49), *P* = 0.01] exhibited significantly higher dabigatran C_peak_ compared to AA genotype carriers, as shown in Figure 5.

Additionally, four studies assessed the SNP's association with clinical outcomes, none of which found a significant impact on bleeding or thrombotic events (10, 11, 15, 18).

##### 3.1.2.2 ABCB1

Multiple studies have investigated the impact of *ABCB1* polymorphisms on the PK and clinical outcomes of dabigatran. For the rs2032582 and rs1128503, four studies consistently reported no significant influence on dabigatran PK or clinical outcomes (10, 11, 20, 25).

###### 3.1.2.2.1 ABCB1 rs1045642

For C_trough_, five studies found no significant association with dabigatran trough levels (14, 15, 17, 24, 25). Regarding C_peak_, only one of four studies reported statistically significant findings (15, 17, 24, 25). Specifically, Sychev et al. reported that TT carriers had significantly higher steady-state C_peak_ compared to CC carriers in patients after total knee arthroplasty (*p* < 0.008) (24). A meta-analysis of three of these studies showed no significant effect of this SNP on C_peak_ levels across four subgroups, albeit with substantial heterogeneity (*I*^2^ range: 58%–90%). This heterogeneity was potentially attributable to the findings reported by Sychev et al. (24). Sensitivity analysis excluding this study partially resolved the heterogeneity (*I*^2^ range: 0%–47%), yet still demonstrated no statistically significant association between *ABCB1* rs1045642 and C_peak_ (Supplementary Figure 2). Furthermore, six studies investigated the relationship between this SNP and clinical outcomes (10, 15, 17, 19, 20, 24), with just one demonstrating statistical significance (17). A meta-analysis of four of these studies indicated that rs1045642 did not significantly affect bleeding risk (Supplementary Figure 3).

###### 3.1.2.2.2 ABCB1 rs4148738

Nine studies assessed its association with C_trough_, with one reporting statistical significance (11, 14, 15, 17, 18, 22, 24–26). A meta-analysis of four studies indicated that GA carriers had significantly lower trough levels compared to AA carriers [MD = −13.94, 95% CI (−27.16, −0.71), *P* = 0.04], as shown in Supplementary Figure 4. For C_peak_, among seven studies (11, 15, 17, 22, 24–26), only one showed a significant association (11). A meta-analysis of four studies indicated no significant effect of the SNP on C_peak_, as shown in Supplementary Figure 5. In terms of clinical outcomes, two out of seven studies suggested a potential link with increased bleeding risk (10, 11, 14, 15, 17–19), but meta-analysis of four studies did not demonstrate a significant association (Supplementary Figure 6).

##### 3.1.2.3 Other genes

Xiang et al. conducted the first GWAS in a Chinese population with NVAF treated with dabigatran. Through whole-exome sequencing and correlation analysis, the study identified that carriers of the minor alleles *UBASH3B* rs2276408 and *FBN2* rs3805625 were associated with an increased risk of bleeding, with the most significant associations observed at the 6-month and 12-month visit (16). This study represents the first identification of an association between these two SNPs and the risk of bleeding.

### 3.2 Rivaroxaban

#### 3.2.1 Pharmacokinetics and pharmacodynamics

Rivaroxaban is rapidly absorbed following oral administration, achieving C_peak_ within 2–4 h (1, 2). Its bioavailability is high but dose-dependent and significantly influenced by food intake. The absolute bioavailability of a 10 mg dose is 80%–100% regardless of food, while for a 20 mg dose, it decreases to 66% under fasting conditions. Therefore, co-administration with food is recommended for the 15 mg and 20 mg doses to enhance bioavailability and systemic exposure (2, 56). Rivaroxaban is highly protein-bound (92%–95%). It undergoes clearance through both hepatic metabolism and renal excretion. Two-thirds of the administered dose is metabolized in the liver to inactive metabolites by cytochrome P450 subtypes 3A4, 3A5, and 2J2, subsequently eliminated equally in urine and feces. The remaining one-third is excreted unchanged in the urine, facilitated by active transporters P-gp and breast cancer resistance protein (BCRP, encoded by *ABCG2*) (58). The terminal half-life is 7–11 h in young subjects (average 9 h) and is prolonged to 11–13 h in the elderly, primarily due to age-related decline in renal clearance (62, 63). Concomitant use with potent dual inhibitors of P-gp and CYP3A4 (e.g., ketoconazole, ritonavir) increases rivaroxaban exposure and bleeding risk and should be undertaken cautiously. Conversely, potent dual inducers (e.g., rifampin, carbamazepine) may reduce exposure, potentially leading to therapeutic failure (8, 58).

#### 3.2.2 Pharmacogenetics of rivaroxaban

##### 3.2.2.1 ABCB1

###### 3.2.2.1.1 ABCB1 rs1045642

**Figure 6:**
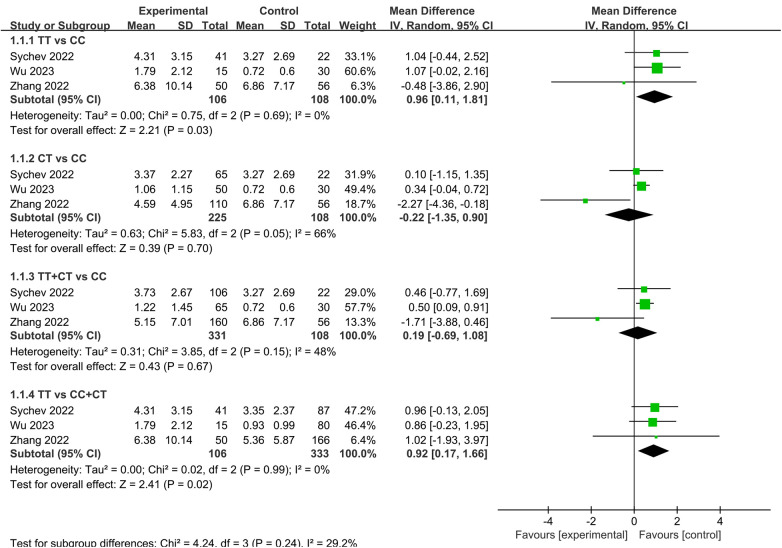
Forest plots illustrating the association between *ABCB1* rs1045642 and C_trough_/D of rivaroxaban.

Ten studies have investigated the association between *ABCB1* rs1045642 and the PK of rivaroxaban (27–30, 32, 34, 37, 39–41). Seven focused on C_trough_, none of which found significant associations. However, our meta-analysis, which included three of these studies, revealed a significant effect of this SNP on dose-adjusted C_trough_ (C_trough_/D) in both homozygous and recessive genetic model subgroups. Specifically, carriers of the TT genotype exhibited significantly higher C_trough_/D compared to both CC genotype carriers and C allele carriers [MD = 0.96, 95% CI [0.11, 1.81], *P* = 0.03; MD = 0.92, 95% CI [0.17, 1.66], *P* = 0.02, respectively], as shown in Figure 6. Regarding C_peak_, among four studies, only Ain et al. reported a statistically significant finding, showing reduced peak and steady-state plasma concentrations in carriers of the CC and CT genotypes relative to TT homozygotes (34). One study on AUC showed no significant association (39).

**Figure 7:**
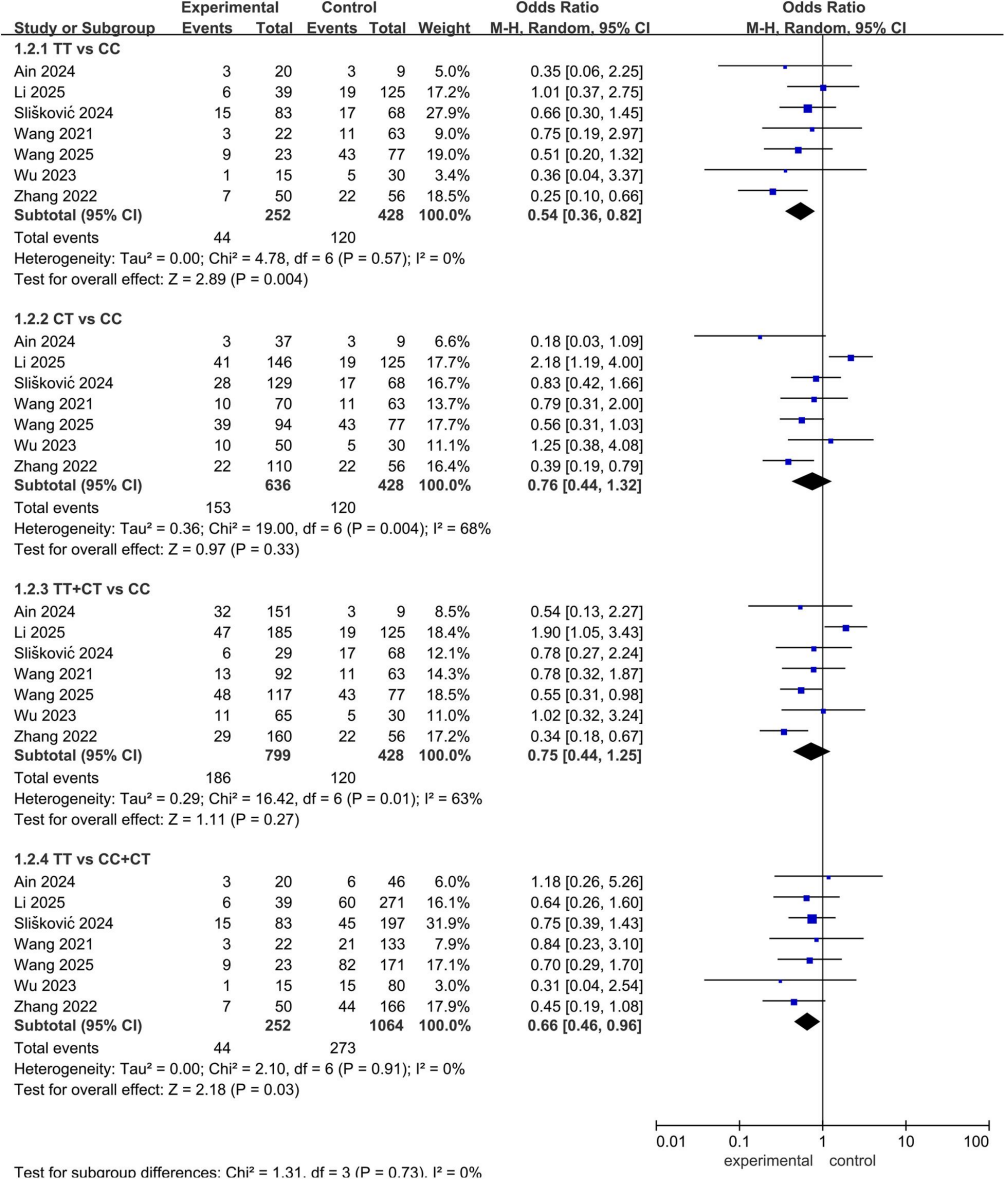
Forest plots illustrating the association between *ABCB1* rs1045642 and bleeding rate of rivaroxaban.

A total of eleven studies assessed the relationship between this SNP and bleeding events (10, 20, 21, 27, 28, 30, 32, 34, 36–38), with only one reporting a significant association (28). Our meta-analysis, synthesizing data from seven of these studies, demonstrated a significant association with bleeding events. The pooled results indicated that TT genotype carriers had a significantly lower risk of bleeding compared to CC carriers and C allele carriers [OR = 0.54, 95% CI (0.36, 0.82), *P* = 0.004; OR = 0.66, 95% CI (0.46, 0.96), *P* = 0.03, respectively], as shown in Figure 7.

For thrombotic events, three studies presented inconsistent findings. Lahteenmaki et al. initially reported that the T allele was associated with a reduced risk of thromboembolic events in a Finnish European population [HR = 0.42, 95%CI (0.18, 0.98)] (10), which was supported by Wang et al. in a Chinese cohort of 371 individuals [HR = 0.19, 95%CI (0.07, 0.56)] (20). However, another recent study indicated an opposing trend, with the TT genotype associated with an increased risk of thromboembolism relative to the CC genotype [RR = 3.48, 95% CI (1.02, 11.85)] (21).

###### 3.2.2.1.2 ABCB1 rs4148738

A total of eight studies investigated the association between *ABCB1* rs4148738 and rivaroxaban PK (27–30, 32, 34, 37, 40). Six studies focused on C_trough_, with only two demonstrating statistical significance (28, 32). Three studies were included in the meta-analysis, which showed no significant effect of this SNP on C_trough_/D across four subgroups, as shown in Supplementary Figure 7. Of the four studies examining C_peak_, only Ain et al. reported significant findings. Their study demonstrated that, compared to GG genotype carriers, AA genotype carriers exhibited significantly lower peak and steady-state concentrations, while GA genotype carriers also showed significantly reduced C_peak_ (34). Additionally, nine studies evaluated the association between this SNP and bleeding risk (21, 27, 28, 30, 32, 34, 36–38), three of which reported statistically significant differences (21, 28, 38). Seven studies were included in the meta-analysis. The pooled results indicated no significant association between *ABCB1* rs4148738 and bleeding risk (Supplementary Figure 8). Substantial heterogeneity was observed across the four genetic models (*I*^2^ range: 45%–81%), and sensitivity analysis failed to identify any single study as the primary source of this variability. Therefore, the findings should be interpreted with caution. Well-designed studies with larger sample sizes are warranted to validate these results.

###### 3.2.2.1.3 *ABCB1* rs1128503

Six studies investigated the association between *ABCB1* rs1128503 and the PK of rivaroxaban (27, 32, 34, 37, 39, 41). Among the four studies focusing on C_trough_, only one found a significant association. Wang et al. observed that CC carriers had significantly lower C_trough_ than TT carriers in a Chinese Mongolian population (27). Two studies focused on C_peak_, with Ain et al. reporting significantly lower peak and steady-state concentrations in CC and CT genotype carriers within a Pakistani cohort (34). One study evaluated AUC, showing no significant association (39). Furthermore, nine studies assessed the relationship between this SNP and clinical outcomes, none of which demonstrated statistical significance (10, 20, 21, 27, 32, 34, 36–38). The meta-analysis incorporating six of these studies revealed that no significant association between *ABCB1* rs1128503 and bleeding risk, as shown in Supplementary Figure 9.

###### 3.2.2.1.4 *ABCB1* rs2032582

Five studies explored the association between *ABCB1* rs2032582 and rivaroxaban PK (30, 34, 37, 39, 41). Of three studies on C_trough_, none found a statistically significant association. Regarding C_peak_, two of three studies reported significant but discordant results. Zhang et al. identified higher dose-adjusted C_peak_ in G allele carriers (30), whereas Ain et al. observed the opposite trend, with reduced peak and steady-state concentrations in GG carriers and decreased C_peak_ in GT carriers (34). One study examined AUC without significant findings (39). For clinical outcomes, eight studies evaluated this SNP (10, 20, 21, 30, 34, 36–38), with one showing a statistically significant association (30).The meta-analysis of four included studies revealed that no significant association between *ABCB1* rs4148738 and bleeding risk (Supplementary Figure 10).

###### 3.2.2.1.5 Other loci

Wu et al. were the first to report an association between the *ABCB1* rs4728709 polymorphism and rivaroxaban PKs. Their study demonstrated that patients carrying the A allele had significantly lower C_trough_/D than the GG wild-type carriers (32). However, this PK difference did not result in a statistically significant increase in bleeding risk. To date, this remains the only study investigating this polymorphism, thus offering novel insights into the genetic factors underlying interindividual variability in rivaroxaban PKs.

##### 3.2.2.2 ABCG2, CYP3A4/5, CYP2J2

Six studies examined the effects of *ABCG2* (rs2231142, rs2231137) on the PK and clinical outcomes of rivaroxaban, with none reporting significant associations. Our meta-analysis of *ABCG2* rs2231142 also showed no significant link with bleeding risk (Supplementary Figure 11). A total of eleven studies evaluated the influence of polymorphisms in *CYP3A5* [*3 (rs776746)], *CYP3A4* [*22 (rs35599367), *1B (rs2740574), rs2242480, rs2246709, rs3735451, rs4646440, rs4646437], *CYP2J2* [*7 (rs890293), rs11572325] and *CYP2C19* [*2 (rs4244285) and *17 (rs12248560)] on rivaroxaban PK and clinical outcomes (20, 29, 30, 32, 34–38, 40, 41). Except for three studies, no significant associations have been established between these gene variants and rivaroxaban response. Notably, in an exploratory analysis by Campos-Staffico et al., carriers of the *CYP3A5* rs776746 G allele were associated with a significantly lower bleeding risk (HR: 0.189, 95% CI: 0.070–0.509) (38). In 2024, Li et al. reported that patients with mutant genotypes of *CYP3A4* (rs2242480, rs2246709, and rs3735451) and *CYP3A5* (rs776746) demonstrated higher C_trough_ and prolonged prothrombin time (PT) compared to those possessing wild-type genotypes. Furthermore, minor alleles of *CYP3A4* (rs3735451 and rs2246709) were linked to an increased incidence of minor bleeding events (35). Subsequently, Ain et al. observed that both homozygous and heterozygous carriers of the *CYP3A5* rs776746 G allele exhibited lower plasma concentrations of rivaroxaban compared with wild-type individuals (34). Meta-analyses further indicated no significant impact of *CYP3A5* rs776746 or *CYP2J2* rs890293 on bleeding events. Although substantial heterogeneity was observed across two genetic models for *CYP2J2* rs890293 (*I*^2^ range: 69%–72%), sensitivity analysis excluding the study by Li et al. (37) completely eliminated this heterogeneity (*I*^2^ = 0%). Nevertheless, no statistically significant association was detected (Supplementary Figures 12, 13).

##### 3.2.2.3 Other genes

Beyond the polymorphisms in drug transporters and metabolizing enzymes discussed above, three studies have explored other genetic loci affecting rivaroxaban outcomes. Using whole-exome sequencing, Zhao et al. initially identified that homozygous carriers of *ABCA6* rs7212506 and *AKR7A3* (rs1738023, rs1738025) had an increased risk of bleeding events (31). Applying a similar methodology, Xiang et al. reported an association between *SUSD3* rs76292544 and 12-month bleeding risk (33). However, evidence remains conflicting. A recent study by Li et al. involving 310 patients corroborated the finding for *SUSD3*, indicating that the TT genotype of rs76292544 constitutes a risk factor for bleeding. In contrast, it failed to replicate the initial associations for *ABCA6* rs7212506 and *AKR7A3* (rs1738023, rs1738025) (37).

### 3.3 Apixaban

#### 3.3.1 Pharmacodynamics and pharmacokinetics

Apixaban is primarily absorbed in the small intestine via passive diffusion, with 50% oral bioavailability unaffected by food (56). C_peak_ is achieved within 3–4 h, with 80% plasma protein binding and a terminal half-life of 12 h (64). The parent drug is the major circulating moiety. It is metabolized in the liver to a limited extent (about 25%), predominantly by CYP3A4/5 isoenzyme, with minor contributions from CYP1A2, CYP2C8, CYP2C9, CYP2C19, and CYP2J2 isoenzymes, and SULT1A1 and SULT1A2 sulfotransferases, mainly SULT1A1. O-demethyl apixaban sulfate is the prominent inactive metabolite (65). Elimination includes multiple pathways, 27% excreted unchanged through the kidneys, while the remainder (73%) eliminated in feces as parent or metabolites (1, 65). Although apixaban exhibits relatively low renal dependency, dose adjustment must strictly adhere to label-specified criteria based on renal function. For example, in patients with NVAF, a dose reduction to 2.5 mg twice daily is recommended if at least two of the following are present: age ≥80 years, body weight ≤60 kg, or serum creatinine ≥1.5 mg/dL. The same dose reduction applies to patients with a creatinine clearance of 15–29 mL/min (66). The use of apixaban in end-stage renal disease (CrCl <15 mL/min) or in patients on hemodialysis is not recommended by the European Medicines Agency, while the U.S. Food and Drug Administration permits use under specific conditions with close monitoring (67). Apixaban is also a substrate for P-gp and BCRP (58). Co-administration with potent dual inhibitors of CYP3A4 and P-gp (e.g., ketoconazole) can double the mean plasma concentration, necessitating caution. Conversely, potent dual inducers (e.g., rifampicin) can decrease plasma levels, potentially resulting in insufficient anticoagulation (1, 2).

#### 3.3.2 Pharmacogenetics

##### 3.3.2.1 ABCB1

**Figure 8:**
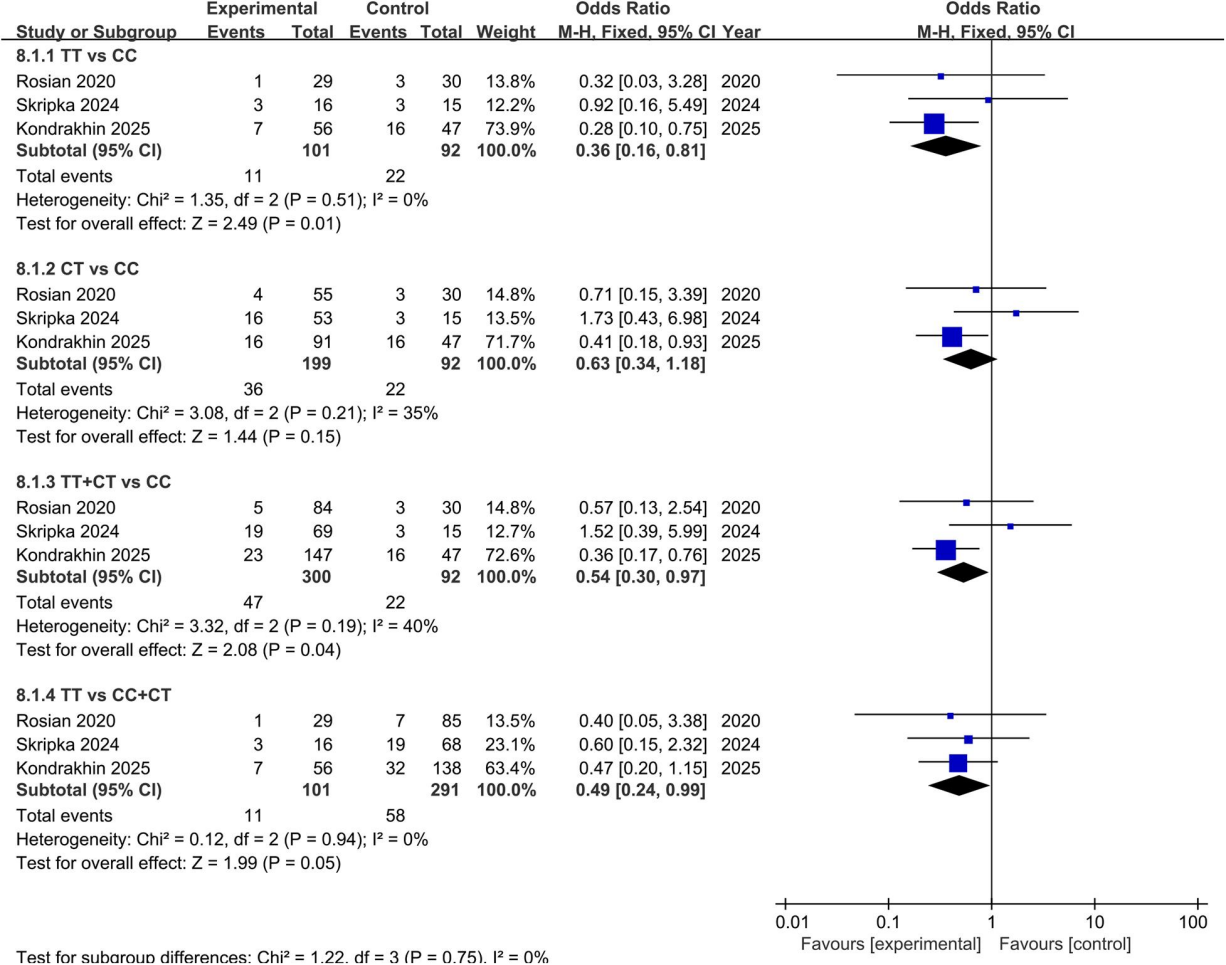
Forest plots illustrating the association between *ABCB1* rs1045642 and bleeding rate of apixaban.

A total of ten studies investigated the influence of rs1045642 on the PK or clinical outcomes of apixaban (10, 19, 38, 39, 42–47). Among these, seven studies focusing on PK revealed no significant associations, while six studies assessing bleeding risk consistently reported no effect except for a recent investigation by Kondrakhin et al. (44), which linked the C allele to increased bleeding risk. Three studies were included in the meta-analysis. The pooled results demonstrated that, compared with the CC wild-type genotype, both TT genotype carriers [OR = 0.36, 95% CI (0.16, 0.81), *P* = 0.01] and T allele carriers [OR = 0.54, 95% CI (0.30, 0.95), *P* = 0.04] had a significantly reduced risk of bleeding. Additionally, TT homozygotes exhibited a significantly lower bleeding risk compared to C allele carriers [OR = 0.49, 95% CI (0.24, 0.99), *P* = 0.05], as shown in Figure 8.

For rs4148738, six studies were identified. Four reported no significant findings (19, 38, 43, 47), whereas two studies yielded statistically significant results. Dimatteo et al. observed lower C_peak_ in AA genotype carriers (23). Additionally, Lähteenmäki et al. reported in a real-world retrospective analysis that the A allele was associated with reduced bleeding risk (10).

Regarding rs1128503 and rs2032582, all six available studies consistently indicated no significant effect of these variants on the PK or clinical outcomes of apixaban (10, 38, 39, 42, 44, 45).

##### 3.3.2.2 ABCG2

Among the five studies investigating the impact of *ABCG2* rs2231142 on PK or clinical outcomes, all three PK-focused studies reported consistent and significant findings. In Japanese patients, the C allele was associated with a higher average oral clearance (45). Similarly, Caucasian patients with the A allele have exhibited increased C_trough_ and C_peak_ (46). A genome-wide association study further corroborated these findings, identifying rs2231142 as the key variant associated with an increased AUC as well as higher peak and trough levels (42). In contrast, the remaining three studies focusing on clinical outcomes did not reveal statistically significant associations (10, 38, 42). Additionally, another common *ABCG2* variant, rs2231137, showed no significant relationship with the PK or clinical outcomes of apixaban (42, 46).

##### 3.3.2.3 CYP3A4/5, CYP2J2

Among seven studies evaluating the influence of *CYP3A5* rs776746 on apixaban PK and clinical outcomes (10, 38, 42–46), five involved clinical outcomes and six involved PK. Two of these studies reported statistically significant findings. Ueshima et al. revealed that *CYP3A5* rs776746 G allele was associated with reduced oral clearance in Japanese patients (45). In an exploratory analysis adjusted for confounders, Campos-Staffico et al. found significantly higher bleeding risk in *CYP3A5* rs776746 G allele carriers [HR = 4.107, 95% CI (1.364, 12.364), *P* = 0.012] (38). Additionally, no significant associations were identified in five studies examining *CYP3A4* [*22 (rs35599367), *1B (rs2740574)] or *CYP2J2* *7 (rs890293) with apixaban PK or clinical outcomes (10, 42–44, 46).

### 3.4 Edoxaban

#### 3.4.1 Pharmacokinetics and pharmacodynamics

Edoxaban is mainly absorbed in the proximal small intestine, with the colon contributing only about 13% to total absorption (58, 68). It exhibits an oral bioavailability of 62% and reaches C_peak_ within 1–2 h (68, 69). Food intake delays absorption but has minimal and clinically insignificant impact on overall exposure (70, 71). The drug is approximately 55% bound to plasma proteins. Edoxaban is metabolized into various metabolites, including the primary active metabolite M-4 (formed via CES1-mediated hydrolysis), which accounts for less than 10% of total drug exposure and exhibits 80% plasma protein binding (72). Other metabolic pathways involve hydrolysis (M1), glucuronidation (M3) and oxidation by CYP3A4/5 (M5, M6, M8) (56, 73). Both edoxaban and M4 are substrates for P-gp and organic anion transporter protein 1B1 (OATP1B1) encoded by *SLCO1B1*, respectively. The elimination half-life ranges from 10 to 14 h. Renal excretion of unchanged drug accounts for approximately 50% of the eliminated dose, while the remainder is eliminated via hepatic metabolism and biliary excretion (58, 73). A 50% dose reduction is recommended for patients with moderate to severe renal impairment (creatinine clearance 15–50 mL/min), low body weight (≤60 kg), or those concomitantly using specific P-gp inhibitors (e.g., cyclosporine, dronedarone). Of note, edoxaban is not recommended for use in patients with NVAF and a CrCl >95 mL/min, as it was associated with reduced efficacy compared to warfarin in clinical trials (74–76). For these patients, another anticoagulant drug should be used.

#### 3.4.2 Pharmacogenetics

Among four studies examining the influence of genetic polymorphisms on edoxaban, three consistently indicated that *ABCB1* (rs1045642, rs1128503, rs2032582) and *CYP3A5* (rs776746) do not significantly affect edoxaban PK or clinical outcomes (20, 48, 49). In contrast, *SLCO1B1* polymorphisms may impact edoxaban PK and bleeding risk. For instance, Nakagawa et al. associated the *SLCO1B1* rs4149056 C allele with increased exposure to the metabolite M4 (48). Han et al. demonstrated that the C alleles of rs4149056 and rs4149057 significantly raise bleeding risk, while the GG genotype of rs2306283 offers protection (9). Wang et al. noted a trend linking the rs4149056 C allele to elevated bleeding risk in a Chinese population [HR = 2.39, 95% CI (0.99, 5.77), *P* = 0.052] (20). Furthermore, the CC genotype of *ABCB1* rs3842 was independently associated with increased bleeding risk (9).

## 4 Discussion

This review included 39 studies, synthesizing the existing evidence on the pharmacogenomics of DOACs and highlighting interindividual differences in drug exposure and clinical outcomes attributed to genetic polymorphisms. Nineteen studies were incorporated into our meta-analysis, which revealed significant effects of *CES1* (rs2244613, rs8192935) and *ABCB1* rs4148738 on dabigatran PK and bleeding risk, *ABCB1* (rs1045642, rs1128503) on rivaroxaban PK and bleeding risk, and *ABCB1* rs1045642 on apixaban bleeding risk.

Importantly, due to the distinct pharmacokinetic pathways of each DOAC, we conducted separate analyses for each drug. This approach minimized clinical heterogeneity and ensures that the reported associations are drug-specific. Consequently, findings for one DOAC should not be extrapolated to another, even when the same genetic variant is involved.

For dabigatran, *CES1* is the most influential gene, and rs2244613 is a key determinant of bleeding risk. Carriers of the C allele exhibit lower C_trough_ and a correspondingly reduced risk of bleeding. Specifically, both the CC genotype and the CA genotype were associated with a decrease in C_trough_. These findings align with prior results from Li and Aldiban et al. (77, 78), underscoring the pronounced effect of *CES1* rs2244613 on dabigatran exposure and its potential role in mediating bleeding or thrombotic complications through PK pathways. Additionally, *CES1* rs8192935 and *ABCB1* rs4148738 also influence dabigatran C_trough_ and C_peak_. Homozygous GG carriers of *CES1* rs8192935 show higher C_trough_ and C_peak_, and G allele carriers generally have elevated peak levels. For *ABCB1* rs4148738, GA carriers demonstrate lower C_trough_ compared to GG carriers, consistent with earlier reports by Aldiban et al. (78). However, these significant effects on PK have not translated into clear differences in clinical outcomes, indicating limited clinical utility at present.

The distribution of the *CES1* alleles varies significantly among different populations. The C allele frequency at the rs2244613 locus is about 57.1% to 63.1% in Asians, compared to only 17.8% to 27.5% in Caucasians (10, 15, 24, 25). Similarly, the G allele frequency at the rs8192935 locus is observed at 22.8% to 38.8% among Asians, whereas it ranges from 67% to 71.5% in Caucasians (10, 11, 17, 25). Despite substantial interethnic variations in allele distribution, the impact of *CES1* genetic variants on PK and clinical outcomes remains consistently significant across populations.

The efficacy and safety of rivaroxaban are significantly modulated by *ABCB1* polymorphisms, with the rs1045642 variant playing a particularly prominent role. *In vitro* and *in vivo* studies have demonstrated that the T allele can downregulate *ABCB1* mRNA levels, reduce intestinal P-gp expression, and potentially affect its function through altered protein folding (79–81), thereby potentially increasing rivaroxaban plasma concentrations. Consistent with this, Xie et al. and Shatalova et al. reported that carriers of the CC genotype show lower AUC from time 0 to infinity and dose-adjusted residual concentrations compared to T allele carriers (82, 83). Our meta-analysis similarly revealed lower C_trough_/D values in CC genotype and C allele carriers relative to AA homozygotes.

However, the CC genotype and C allele carriers were associated with a higher risk of bleeding, contradicting the conventional pharmacokinetic-pharmacodynamic relationship. This suggests that rs1045642 may modulate bleeding through pathways independent of plasma concentration. This SNP is in strong linkage disequilibrium with rs1128503 and rs2032582, forming major haplotypes such as 1236C-2677G-3435C and 1236T-2677T-3435T (84), whose combined effects may obscure the true impact of individual SNPs. Moreover, linkage with unobserved functional variants in the region could also affect P-gp activity and consequently alter bleeding risks (85). Notably, the allele frequency of rs1045642 varies considerably across ethnicities (84), and its association with clinical outcomes may be confounded by population-specific genetic backgrounds, concomitant medications, and comorbidities, thus warranting cautious interpretation. To date, the effect of this SNP on thrombotic events remains controversial. Further research is needed in the future to clarify its complex regulatory mechanism in the treatment with rivaroxaban.

In clinical practice, established factors such as renal or hepatic impairment, concomitant use of interacting medications, low body weight, and advanced age are known to significantly elevate bleeding risk in rivaroxaban users (12). These potent clinical confounders may obscure, confound, or amplify the independent effect of *ABCB1* polymorphisms, potentially introducing bias into the association analyses. Consequently, current evidence does not support routine *ABCB1* testing in clinical practice to guide rivaroxaban therapy. In contrast, polymorphisms including *ABCG2* rs2231142, *CYP3A5* rs776746, and *CYP2J2* rs890293 have no significant influence on bleeding events. The emergence of novel loci such as *SUSD3* rs76292544 offers fresh insights into bleeding risk mechanisms, calling for further large-scale investigation.

Similar to rivaroxaban, *ABCB1* rs1045642 is associated with a reduced bleeding risk in patients using apixaban. Individuals carrying the TT genotype or T allele exhibit a significantly lower risk of bleeding. Meanwhile, the A allele of *ABCG2* rs2231142 markedly raises apixaban exposure without affecting clinical outcomes. In contrast, the other polymorphisms of *ABCB1*, *CYP3A4/5* and *CYP2J2* have a relatively small and heterogeneous impact on apixaban. It is noteworthy that the PKs of apixaban are influenced by multiple clinical factors. Renal function is consistently identified as the primary determinant (39, 45, 47), while factors such as sex, age, heart failure, and P-gp phenotypic activity are also correlated with drug exposure levels (23, 39, 46, 47).

Compared to other DOACs, edoxaban has been less extensively studied, with data predominantly derived from Asian populations. The clearance of its active metabolite M-4 is primarily mediated by the OATP1B1 transporter, encoded by the *SLCO1B1* gene. Current evidence suggests that genetic polymorphisms in *SLCO1B1*, particularly the rs4149056 C allele, may lead to increased M-4 exposure and elevated bleeding risk. This represents a distinct metabolic pathway not commonly shared by other DOACs, highlighting the need for further investigation.

Furthermore, several meta-analyses in this review were based on a limited number of studies or small sample sizes, particularly for SNPs with non-significant findings. Therefore, these null results should be interpreted with caution, as they may reflect insufficient statistical power rather than a true absence of association. Larger, well-powered studies are needed to confirm or refute these findings.

To date, neither PharmGKB nor the Clinical Pharmacogenetics Implementation Consortium (CPIC) has issued formal guidelines for genotype-guided dosing of DOACs. On PharmGKB, genetic polymorphisms such as *CES1* and *ABCB1* are assigned a level 3 evidence (low evidence) rating for DOACs, reflecting suggestive but clinically insufficient evidence. Given the observational nature of the included studies and the substantial heterogeneity in allele annotation, study populations, and endpoint definitions, the current evidence should be considered hypothesis-generating rather than practice-changing. Therefore, routine genotyping for DOACs is not supported in clinical practice. To advance toward clinical implementation, future research must prioritize: (1) replication in diverse ancestral populations, (2) standardization of pharmacokinetic sampling and outcome definitions, and (3) prospective validation of genotype–outcome associations with rigorous adjustment for clinical confounders.

## 5 Limitations

This systematic review and meta-analysis has several limitations. First, there was heterogeneity among the included studies in design, sample size, definition of clinical endpoints, and statistical methods. Only 19 studies were eligible for meta-analysis, and some analyses were based on small numbers of studies or participants, limiting statistical power to detect true associations. Second, although we harmonized allele annotations, inconsistencies in original reporting may have introduced residual misclassification. Third, key clinical confounders (e.g., renal function, concomitant medications, age) were not uniformly adjusted for across studies, leaving potential residual confounding. Fourth, most studies did not account for population stratification, and the underrepresentation of non-European and non-Asian populations limits generalizability. Fifth, despite using “All Fields” searches, some studies may have been missed if they used terminology not captured by our search terms; restricting to English-language publications may also have introduced language bias.

## 6 Conclusion

This systematic review synthesizes current evidence on the pharmacogenomics of DOACs. Several SNPs, including *CES1* (rs2244613, rs8192935), *ABCB1* rs1045642, *ABCG2* rs2231142, and *SLCO1B1* rs4149056, were consistently associated with variability in DOAC pharmacokinetics or clinical outcomes. However, due to heterogeneity in allele annotation, study populations, and endpoint definitions, along with potential residual confounding and the predominantly observational nature of the included studies, these findings warrant cautious interpretation. Current evidence does not support routine genotype-guided dosing in clinical practice, and established clinical factors remain the foundation for dose selection and therapeutic management. Future research should prioritize large, multi-ethnic prospective studies with standardized outcome definitions and rigorous control for confounders to validate these genetic markers and evaluate their potential role in personalized anticoagulation therapy.

## Data Availability

The original contributions presented in the study are included in the article/Supplementary Material, further inquiries can be directed to the corresponding author.
