# Supplementary material for: Pharmacogenomics of direct oral anticoagulants: a systematic review and meta-analysis
Source: Front Cardiovasc Med. 2026 Apr 14;13:1792186. doi: 10.3389/fcvm.2026.1792186 (PMC13121062; doi:10.3389/fcvm.2026.1792186)
Supplement: Supplementary file 1 [file Datasheet1.pdf]

frontiers

Supplementary Material

Supplementary Table S1. Literature Search Strategy

<table>
<tr><th>Database</th><th>Restrictions</th><th>Access date</th><th>Search strategy</th><th>No of results</th></tr>
<tr><td>PubMed</td><td>All fields</td><td>29/10/2025</td><td rowspan="4">(dabigatran OR apixaban OR rivaroxaban OR edoxaban) AND

(CES1 OR ABCB1 OR ABCG2 OR SLCO1B1 OR CYP3A4 OR CYP3A5 OR pharmacogenomics OR pharmacogenetics OR gene polymorphism)</td><td>343</td></tr>
<tr><td>Cochrane</td><td>Title/Abstract/ keyword</td><td>29/10/2025</td><td>50</td></tr>
<tr><td>Embase</td><td>All fields</td><td>29/10/2025</td><td>347</td></tr>
<tr><td>Web of Science</td><td>All fields</td><td>29/10/2025</td><td>333</td></tr>
<tr><td colspan="4">Total</td><td>1073</td></tr>
</table>

TABLE S2 The characteristics of the included studies related to dabigatran.

Study ID	Type of study	Participants	Country	Ethnicity	Sample Size	Age	Men/Women
Paré et al. 2013	Prospective cohort	Patients with AF and at least 1 additional risk factor for stroke	Canada	Predominantly Caucasian	1694	71.8 ± 7.5	1163/531
Sychev et al. 2020	Prospective cohort	Patients with AF and stage 3 CKD	Russia	Caucasian	96	75 (51-89)	39/57
Ji et al. 2021	Prospective cohort	Patients with NVAF	China	Han Chinese	198	63.3 ± 9.3	120/78
Xiang et al. 2022	Prospective cohort	Patients with NVAF	China	Han Chinese	170	72.0(65.8,79.0)	103/67
Zhu et al. 2022	Prospective cohort	Patients	China	Han Chinese	86	72.15 ± 9.17	49/37
Olšerová et al. 2024	Retrospective cohort	Patients with ischemic stroke	Czech Republic	Caucasian	432	67.56 ± 14.7	244/188
Rosian et al. 2020	Prospective cohort	Patients	Romania	Caucasian	104	70.89 ± 8.85	55/49
Lähteenmäki et al. 2021	Retrospective cohort	Patients	Finland	Caucasian	340	69.8 ± 8.7	178/162
Wang et al. 2025	Retrospective cohort	Patients	China	Han Chinese	183	68.8±10.4	106/77
Dimatteo et al. 2016	Prospective cohort	Outpatients	Italy	Caucasian	92	72.0 (52-92)	51/41
Sychev et al. 2018	Prospective cohort	Patients after total knee arthroplasty	Russia	Caucasian	60	62.0 (37-81)	2/58
Abdrakhmanov et al. 2024	Prospective cohort	Patients with NVAF	Kazakhstan	Kazakh	150	58.9 ± 10.3	90/60
Cumitini et al. 2024	Prospective cohort	Patients with AF	Italy	Caucasian	100	77(68-82)	63/37

Abbreviations: AF, atrial Fibrillation; NAVF, non-valvular atrial fibrillation; CKD, Chronic kidney disease.

TABLE S3 The characteristics of the included studies related to rivaroxaban.

Study ID	Type of study	Participants	Country	Ethnicity	Sample Size	Age	Men/Women
Wang et al. 2021	Retrospective cohort	Patients with NVAF	China	Mongolian	155	71.98±10.72	81/74
Sychev et al. 2022	Cross-sectional	Patients with NVAF	Russia	Caucasion	128	87.5 (83.0–90.0)	NR
Zhang et al. 2022	Prospective cohort	Patients with NVAF	China	Han Chinese	216	72.2	102/114
Zhao et al. 2022	Retrospective case-control	Patients	China	Han Chinese	45	80.95	22/23
Wu et al. 2023	Prospective cohort	Patients with NVAF	China	Han Chinese	95	65.8±12.5	56/39
Xiang et al. 2023	Prospective cohort	Patients with NVAF	China	Han Chinese	257	68.9±10.4	133/124
Ain et al. 2024	Prospective cohort	Patients with NVAF	Pakistan	Pakistani	66	70.24±6.04	NR
Li et al. 2024	Prospective cohort	Patients with NVAF	China	Han Chinese	165	66.83±11.49	92/73
Slšković et al. 2024	Prospective case-control	Patients	Croatia	Caucasion	280	65(26-98)	172/108
Li et al. 2025	Prospective cohort	Patients with VTE	China	Han Chinese	310	65.18±14.53	176/134
Wang et al. 2025	Prospective cohort	Patients with NVAF	China	Han Chinese	228	72.3±10	140/88
Lähteenmäki et al. 2021	Retrospective cohort	Patients	Finland	Caucasian	999	69.6±9.8	501/498
Campos-Staffico et al. 2022	Retrospective cohort	Patients with NVAF	America	Caucasian	1040	67.5±13.2	704/336
Wang et al. 2025	Retrospective cohort	Patients with NVAF	China	Han Chinese	371	70.3±11.3	211/160
Lenoir et al. 2022	Prospective cohort	Patients with AF or VTE	Switzerland	Caucasian	135	71.1±12.1	89/46
Sychev et al. 2019	Prospective cohort	Patients*	Russia	Caucasian	78	59±11	22/56
Nakagawa et al. 2021	Prospective cohort	Patients with NVAF	Japan	Japanese	86	62.4±10.6	73/13
Sychev et al. 2022	Prospective cohort	Patients with NVAF	Russia	Caucasian	86	67.24±1.01	42/44

Abbreviations: AF, atrial Fibrillation; NAVF, non-valvular atrial fibrillation; VTE, venous thromboembolism.

TABLE S4 The characteristics of the included studies related to apixaban.

Study ID	Type of study	Participants	Country	Ethnicity	Sample Size	Age	Men/Women
Attelind et al. 2022	Prospective cohort	Patients	Sweden	Caucasian (94%)	1,325	69 ± 9	887/438
Skripka et al. 2024	Prospective cohort	Patients with NVAF	Russia	Caucasian	84	71.6 ± 8.33	29/55
Kondrakhin et al. 2025	Cross-sectional	Patients with NVAF	Russia	Caucasian	197	83 ± 8	65/132
Roşian et al. 2020	Prospective cohort	Patients	Romania	Caucasian	114	70.97 ± 9.24	58/56
Lähteenmäki et al. 2021	Retrospective cohort	Patients	Finland	Caucasian	467	72.3 ± 9.5	228/239
Campos-Staffico et al. 2022	Retrospective cohort	Patients with NVAF	America	Caucasian	1324	69.1±13.6	902/422
Dimatteo et al. 2016	Prospective cohort	Outpatients	Italy	Caucasian	80	76 (38-90)	48/32
Ueshima et al. 2018	Prospective cohort	Patients with AF	Japan	Japanese	81	68.1 (40.5-84.9)	61/20
Gulilat et al. 2020	Prospective cohort	Patients with AF	Canada	Caucasian	358	77.2 ± 10.5	192/164
Roşian et al. 2020	Prospective cohort	Patients with NVAF	Romania	Caucasian	53	70 (65-77)	32/21
Lenoir et al. 2022	Prospective cohort	Patients with AF or VTE	Switzerland	Caucasian	164	74 ± 9.8	101/63

Abbreviations: AF, atrial Fibrillation; NAVF, non-valvular atrial fibrillation; VTE, venous thromboembolism.

Table S5 The characteristics of the included studies related to edoxaban.

Study ID	Type of study	Participants	Country	Ethnicity	Sample Size	Age	Men/Women
Han et al. 2023	Retrospective cohort	Patients	Korea	Korean	159	71	92/67
Wang et al. 2025	Retrospective cohort	Patients with NVAF	China	Han Chinese	166	72.4 ± 9.7	94/72
Nakagawa et al. 2023	Retrospective cohort	Patients with NVAF	Japan	Japanese	152	65.2 ± 10.6	95/57
Ueshima et al. 2025	Retrospective cohort	Patients with AF	Japan	Japanese	131	72.2 (35.2-92.6)	83/48

Abbreviations: AF, atrial Fibrillation; NAVF, non-valvular atrial fibrillation.

Table S6 The nomenclature of single nucleotide polymorphisms and allelic harmonization.

Single nucleotide polymorphism	HGVS nomenclature	Conventional nomenclature	Effect allele
CES1 rs2244613	NC_000016.10: g.55810697G>T	1168-33A>C	C
CES1 rs8192935	NC_000016.10: g.55827882A>G		G
CES1 rs71647871	NC_000016.10: g.55823658C>T	428G>A	A
ABCB1 rs1045642	NC_000007.14: g.87509329A>G	3435C>T	T
ABCB1 rs4148738	NC_000007.14: g.87533733C>T	2482-2236G>A	A
ABCB1 rs1128503	NC_000007.14: g.87550285A>G	1236C>T	T
ABCB1 rs2032582	NC_000007.14: g.87531302A>C/T	2667G>T/A	T/A
ABCB1 rs3842	NC_000007.14: g.87504050T>C		C
ABCB1 rs4728709	NC_000007.14: g.87604285G>A		A
ABCG2 rs2231142	NC_000004.12: g.88131171G>T	421C>A	A
ABCG2 rs2231137	NC_000004.12: g.88139962C>T		T
CYP3A4 rs35599367	NC_000007.14: g.99768693G>A	*22(522-191C>T)	T
CYP3A4 rs2740574	NC_000007.14: g.99784473C>T	*1B(-392A>G)	G
CYP3A4 rs2242480	NC_000007.14: g.99763843C>T		T
CYP3A4 rs2246709	NC_000007.14: g.99768096A>G		G
CYP3A4 rs3735451	NC_000007.14: g.99758352T>C		C
CYP3A4 rs4646440	NC_000007.14: g.99763247G>A		A
CYP3A4 rs4646437	NC_000007.14: g.99767460G>A		A
CYP3A5 rs776746	NC_000007.14: g.99672916T>C	*3(6986A>G)	G
CYP2C19 rs4244285	NC_000010.11: g.94781859G>A	*2(681G>A)	A
CYP2C19 rs12248560	NC_000010.11: g.94761900C>T	*17(-806C>T)	T
CYP2J2 rs890293	NC_000001.11: g.59926822C>A	*7(-76G>T)	T
CYP2J2 rs11572325	NC_000001.11: g.59896030A>T		T
SLCO1B1 rs4149056	NC_000012.12: g.21178615T>C	521T>C	C
SLCO1B1 rs2306283	NC_000012.12: g.21176804A>G	388A>G	G
SLCO1B1 rs4149057	NC_000012.12: g.21178665T>C	571T>C	C
SLCO1B1 rs4149081	NC_000012.12: g.21225087G>A		A
SLCO1B1 rs11045879	NC_000012.12: g.21229685T>C		C
SLCO1B1 rs999278	NC_000012.12: g.21180717C>A		A
SLCO1B1 rs12317268	NC_000012.12: g.21199607A>G		G
SLCO1B1 rs10841753	NC_000012.12: g.21168436T>C		C
SLCO1B1 rs2417957	NC_000012.12: g.21170677C>T		T
SLCO1B1 rs4149042	NC_000012.12: g.21175938T>C		C
SUSD3 rs76292544	NC_000009.12: g.93079458A>T		T
NCMAP rs4553122	NC_000001.11: g.24601069T>C		C
PRF1 rs885821	NC_000010.11: g.70598898G>A		A
PRKAG2 rs12703159	NC_000007.14: g.151807411C>T		T
PRKAG2 rs13224758	NC_000007.14: g.151807372G>A		A
POU2F3 rs2298579	NC_000011.10: g.120299811T>C		C

AKR7A3 rs1738023	NC_000001.11: g.19284747T>C		C
AKR7A3 rs1738025	NC_000001.11: g.19282760T>C		C
ABCA6 rs7212506	NC_000017.11: g.69105577C>T		T
UBASH3B rs2276408	NC_000011.10: g.122808203C>T		T
FBN2 rs3805625	NC_000005.10: g.128278869G>T		T
SULT1A1 rs1042028	NC_000016.10: g.28606193C>T		T

Table S7 The quality assessment of the included case-control studies or cohort studies with Newcastle-Ottawa Scale.

Study ID	Selection				Comparability	Outcome			Scores
	Representativeness of the Exposed Cohort	Selection of the Non Exposed Cohort	Ascertai- nment of Exposure	Demonstration that Outcome of Interest was not Present at Start of Study	Comparability of Cohorts on the Basis of the Design or Analysis	Assess- ment of Outcome	Was Follow up Long Enough for Outcomes to Occur	Adequacy of Follow up of Cohorts	
Paré et al. 2013	1	1	1	1	0	1	1	1	7
Sychev et al. 2020	1	1	1	1	0	1	1	1	7
Ji et al. 2021	1	1	1	1	0	1	1	1	7
Xiang et al. 2022	1	1	1	1	0	1	1	1	7
Zhu et al. 2022	1	1	1	1	0	1	1	0	6
Olšerová et al. 2024	1	1	1	1	1	1	1	1	8
Rosian et al. 2020	1	1	1	1	0	1	1	1	7
Lähteenmäki et al. 2021	1	1	1	1	1	1	1	1	8
Wang et al. 2025	1	1	1	1	2	1	1	1	9
Dimatteo et al. 2016	1	1	1	1	0	1	1	1	7
Sychev et al. 2018	1	1	1	1	0	1	1	1	7
Abdrakhmanov et al. 2024	1	1	1	1	0	1	1	1	7
Cumitini et al. 2024	1	1	1	1	0	1	1	1	7
Wang et al. 2021	1	1	1	1	2	1	1	1	9
Zhang et al. 2022	1	1	1	1	2	1	0	1	8
Zhao et al. 2022	0	1	1	1	1	1	1	1	7
Wu et al. 2023	1	1	1	1	2	1	1	0	8
Xiang et al. 2023	1	1	1	1	0	1	1	1	7
Ain et al. 2024	1	1	1	1	0	1	0	1	6
Li et al. 2024	1	1	1	1	2	1	1	1	9
Slšković et al. 2024	1	1	1	1	2	1	1	1	9
Li et al. 2025	1	1	1	1	1	1	0	1	7
Wang et al. 2025	1	1	1	1	0	1	1	1	7
Campos-Staffico et al. 2022	1	1	1	1	2	1	1	1	9
Lenoir et al. 2022	1	1	1	1	2	1	1	0	8
Sychev et al. 2019	1	1	1	1	0	1	1	0	6
Nakagawa et al. 2021	1	1	1	1	0	1	1	1	7
Sychev et al. 2022	1	1	1	1	0	1	1	1	7
Attelind et al. 2022	1	1	1	1	0	1	1	7	7
Skripka et al. 2024	1	1	1	1	0	1	1	1	7
Dimatteo et al. 2016	1	1	1	1	0	1	1	1	7

Ueshima et al. 2018	1	1	1	1	0	1	1	1	7
Gulilat et al. 2020	1	1	1	1	2	1	1	1	9
Roşian et al. 2020	1	1	1	1	0	1	1	1	7
Han et al. 2023	1	1	1	1	2	1	1	1	8
Nakagawa et al. 2023	1	1	1	1	2	1	1	1	9
Ueshima et al. 2025	1	1	1	1	0	1	1	1	7

Table S8 The quality assessment of the included cross-sectional studies with Agency for Healthcare Research and Quality standards.

Study ID	Define the source of information (survey, record review)	List inclusion and exclusion criteria for exposed and unexposed subjects (cases and controls) or refer to previous publications	Indicate time period used for identifying patients	Indicate whether or not subjects were consecutive if not population-based	Indicate if evaluators of subjective components of study were masked to other aspects of the participants	Describe any assessments undertaken for quality assurance purposes (e.g.,test/retest of primary outcome measurements)	Explain any patient exclusions from analysis	Describe how confounding was assessed and/or controlled	If applicable, explain how missing data were handled in the analysis	Summarize patient response rates and completeness of data collection	Clarify what follow-up, if any, was expected and the percentage of patients for which incomplete data or follow-up was obtained	Scores
Sychev et al. 2022	1	1	1	0	1	0	0	1	0	1	0	6
Kondrakhin et al. 2025	1	1	1	0	1	0	0	1	0	1	0	6

Study or Subgroup	Experimental Mean	Experimental SD	Experimental Total	Control Mean	Control SD	Control Total	Weight	Mean Difference IV, Random, 95% CI
**1.2.1 CC vs AA**								
Abdrakhmanov 2024	110.94	61.32	37	115.03	86.94	43	24.7%	-4.09 [-36.73, 28.55]
Cumitini 2024	93	2.01	5	121.92	67.91	60	56.6%	-28.92 [-46.19, -11.65]
Zhu 2022	89.16	57	20	91.54	48.04	10	18.6%	-2.38 [-41.25, 36.49]
**Subtotal (95% CI)**			**62**			**113**	**100.0%**	**-17.83 [-36.19, 0.53]**
Heterogeneity: Tau² = 77.32; Chi² = 2.72, df = 2 (P = 0.26); I² = 26%								
Test for overall effect: Z = 1.90 (P = 0.06)								
**1.2.2 CA vs AA**								
Abdrakhmanov 2024	94.57	68.26	70	115.03	86.94	43	33.7%	-20.46 [-50.97, 10.05]
Cumitini 2024	132.95	59.96	35	121.92	67.91	60	39.3%	11.03 [-15.24, 37.30]
Zhu 2022	113.09	58.86	28	91.54	48.04	10	26.9%	21.55 [-15.35, 58.45]
**Subtotal (95% CI)**			**133**			**113**	**100.0%**	**3.23 [-20.69, 27.15]**
Heterogeneity: Tau² = 199.05; Chi² = 3.60, df = 2 (P = 0.17); I² = 44%								
Test for overall effect: Z = 0.26 (P = 0.79)								
**1.2.3 CC+CA vs AA**								
Abdrakhmanov 2024	100.28	66.09	107	115.03	86.94	43	32.6%	-14.75 [-43.60, 14.10]
Cumitini 2024	127.96	57.57	40	121.92	67.91	60	44.1%	6.04 [-18.73, 30.81]
Zhu 2022	103.12	58.7	48	91.54	48.04	10	23.3%	11.58 [-22.51, 45.67]
**Subtotal (95% CI)**			**195**			**113**	**100.0%**	**0.56 [-15.89, 17.02]**
Heterogeneity: Tau² = 0.00; Chi² = 1.67, df = 2 (P = 0.43); I² = 0%								
Test for overall effect: Z = 0.07 (P = 0.95)								
**1.2.4 CC vs CA+AA**								
Abdrakhmanov 2024	110.94	61.32	37	102.53	76.36	113	32.3%	8.41 [-15.85, 32.67]
Cumitini 2024	93	2.01	5	125.98	64.99	95	39.8%	-32.98 [-46.17, -19.79]
Zhu 2022	89.16	57	20	107.42	56.41	38	27.8%	-18.26 [-49.01, 12.49]
**Subtotal (95% CI)**			**62**			**246**	**100.0%**	**-15.49 [-42.23, 11.25]**
Heterogeneity: Tau² = 422.26; Chi² = 8.73, df = 2 (P = 0.01); I² = 77%								
Test for overall effect: Z = 1.14 (P = 0.26)								

Test for subgroup differences: Chi² = 3.24, df = 3 (P = 0.36), I² = 7.3%

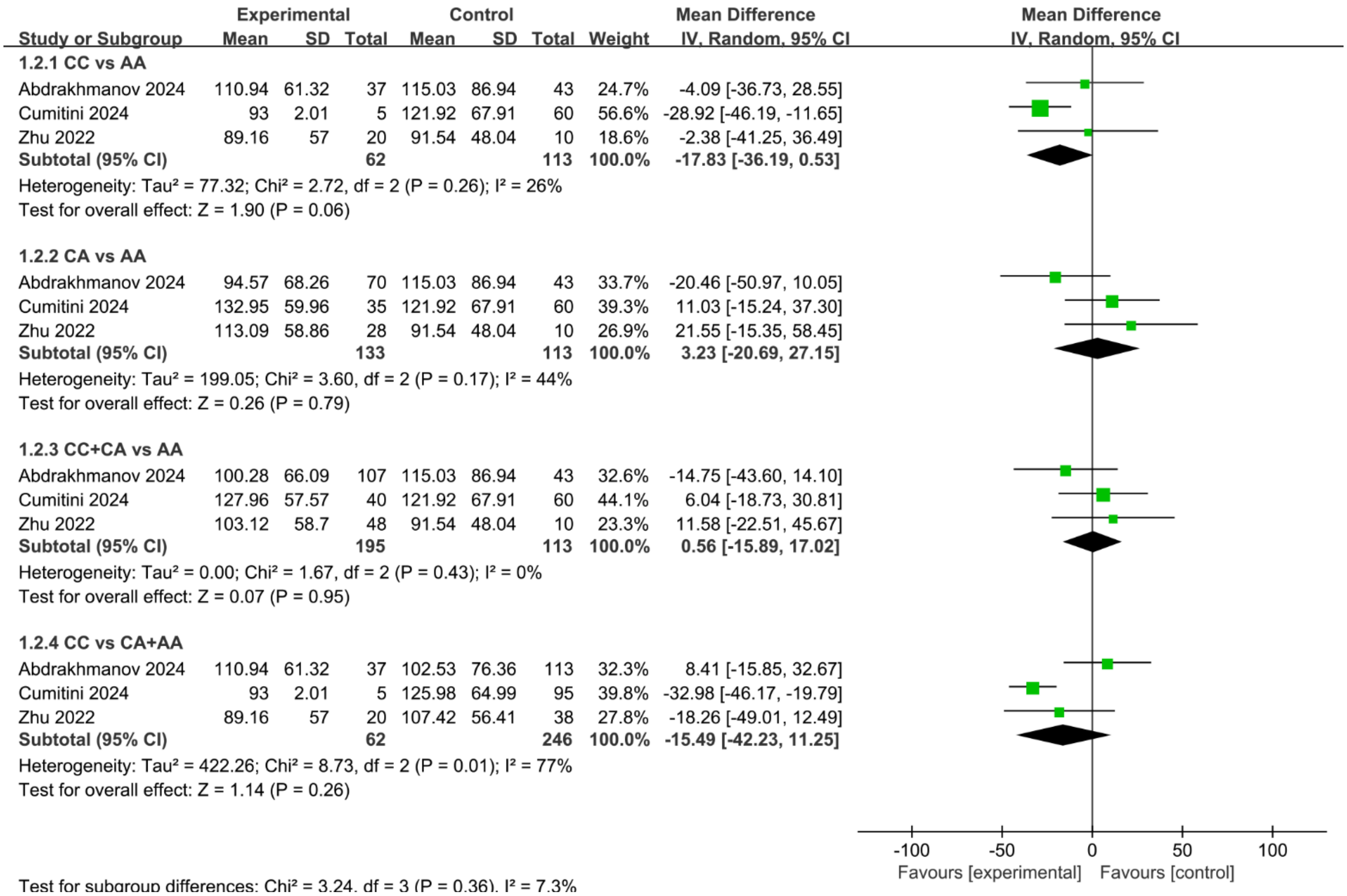

Supplementary Figure S1. Forest plots illustrating the relationship between *CES1* rs2244613 and C_{peak} of dabigatran.

Study or Subgroup	Experimental Mean	Experimental SD	Experimental Total	Control Mean	Control SD	Control Total	Weight	Mean Difference IV, Random, 95% CI	Year
**3.1.1 TT vs CC**									
Sychev 2018	275.5	123.06	16	127.51	79.99	15	0.0%	147.99 [75.36, 220.62]	2018
Zhu 2022	75.55	59.19	11	114.45	58.29	24	42.6%	-38.90 [-80.94, 3.14]	2022
Abdrakhmanov 2024	101.03	73.75	33	103.08	66.4	47	57.4%	-2.05 [-33.57, 29.47]	2024
**Subtotal (95% CI)**			**44**			**71**	**100.0%**	**-17.74 [-53.45, 17.97]**	
Heterogeneity: Tau² = 319.61; Chi² = 1.89, df = 1 (P = 0.17); I² = 47%									
Test for overall effect: Z = 0.97 (P = 0.33)									
**3.1.2 CT vs CC**									
Sychev 2018	189.98	128.97	29	127.51	79.99	15	0.0%	62.47 [0.49, 124.45]	2018
Zhu 2022	99.44	51.85	23	114.45	58.29	24	40.9%	-15.01 [-46.52, 16.50]	2022
Abdrakhmanov 2024	107.26	77.18	70	103.08	66.4	47	59.1%	4.18 [-22.04, 30.40]	2024
**Subtotal (95% CI)**			**93**			**71**	**100.0%**	**-3.67 [-23.82, 16.48]**	
Heterogeneity: Tau² = 0.00; Chi² = 0.84, df = 1 (P = 0.36); I² = 0%									
Test for overall effect: Z = 0.36 (P = 0.72)									
**3.1.3 TT+CT vs CC**									
Sychev 2018	220.39	132.14	45	127.51	79.99	15	0.0%	92.88 [36.94, 148.82]	2018
Zhu 2022	91.71	54.61	34	114.45	58.29	24	43.6%	-22.74 [-52.42, 6.94]	2022
Abdrakhmanov 2024	105.28	75.81	103	103.08	66.4	47	56.4%	2.20 [-21.77, 26.17]	2024
**Subtotal (95% CI)**			**137**			**71**	**100.0%**	**-8.67 [-32.91, 15.57]**	
Heterogeneity: Tau² = 121.56; Chi² = 1.64, df = 1 (P = 0.20); I² = 39%									
Test for overall effect: Z = 0.70 (P = 0.48)									
**3.1.4 TT vs CT+CC**									
Sychev 2018	275.5	123.06	16	168.68	117.52	44	0.0%	106.82 [37.24, 176.40]	2018
Zhu 2022	75.55	59.19	11	107.1	55.16	47	38.1%	-31.55 [-69.92, 6.82]	2022
Abdrakhmanov 2024	101.03	73.75	33	105.62	72.86	117	61.9%	-4.59 [-33.01, 23.83]	2024
**Subtotal (95% CI)**			**44**			**164**	**100.0%**	**-14.86 [-40.52, 10.80]**	
Heterogeneity: Tau² = 66.71; Chi² = 1.22, df = 1 (P = 0.27); I² = 18%									
Test for overall effect: Z = 1.14 (P = 0.26)									

Test for subgroup differences: Chi² = 0.70, df = 3 (P = 0.87), I² = 0%

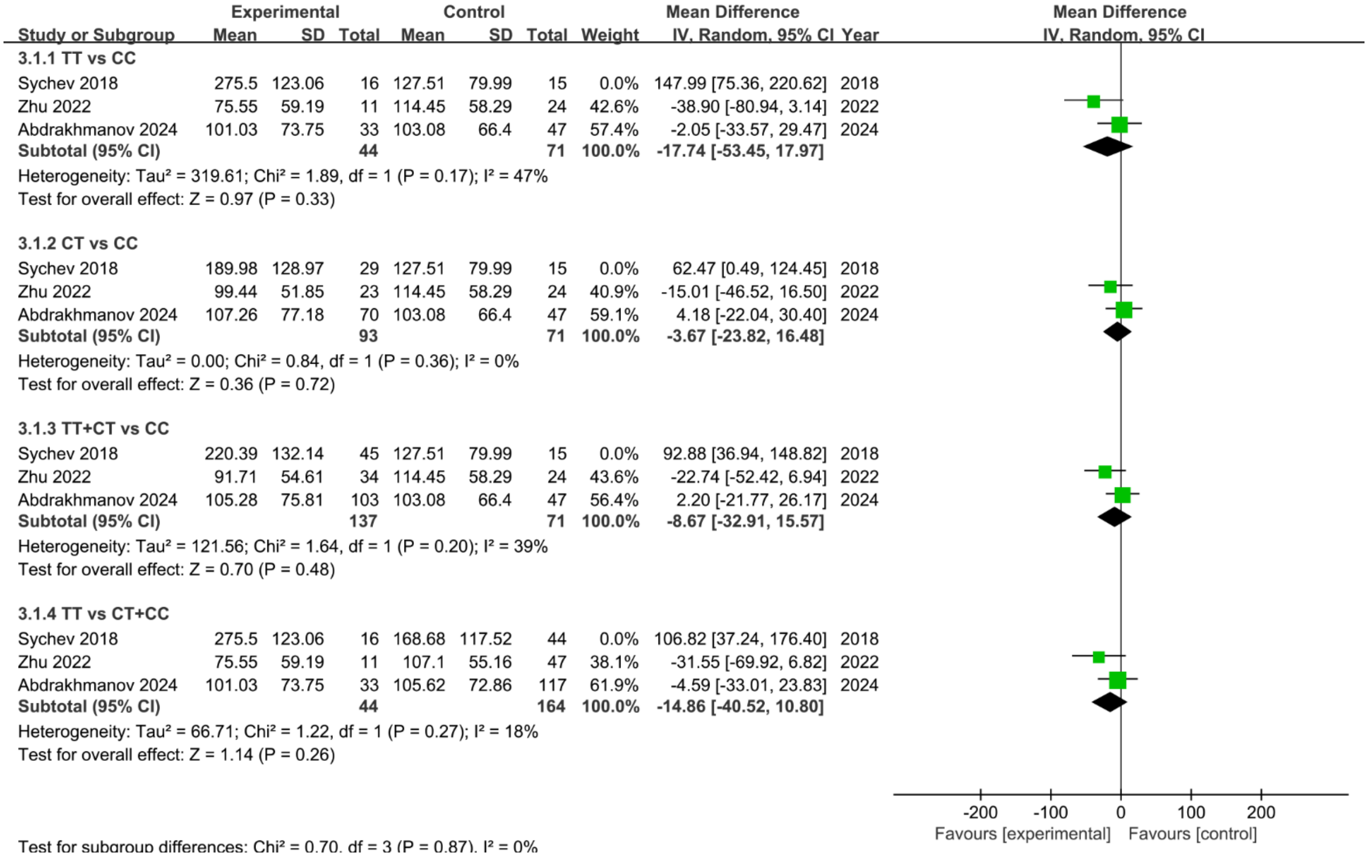

Supplementary Figure S2. Forest plots illustrating the relationship between *ABCB1* rs1045642 and C_{peak} of dabigatran.

Study or Subgroup	log[Odds Ratio]	SE	Weight	Odds Ratio IV, Fixed, 95% CI	Year
Rosian 2020	0.2113	0.8468	8.7%	1.24 [0.23, 6.49]	2020
Sychev 2020	-0.2281	0.7147	12.2%	0.80 [0.20, 3.23]	2020
Ji et al. 2021	0.1235	0.4065	37.8%	1.13 [0.51, 2.51]	2021
Zhu 2022	0.8207	0.389	41.3%	2.27 [1.06, 4.87]	2022
**Total (95% CI)**			**100.0%**	**1.46 [0.89, 2.38]**	

Heterogeneity: $Chi^2 = 2.44$, df = 3 (P = 0.49); $I^2 = 0\%$
Test for overall effect: Z = 1.50 (P = 0.13)

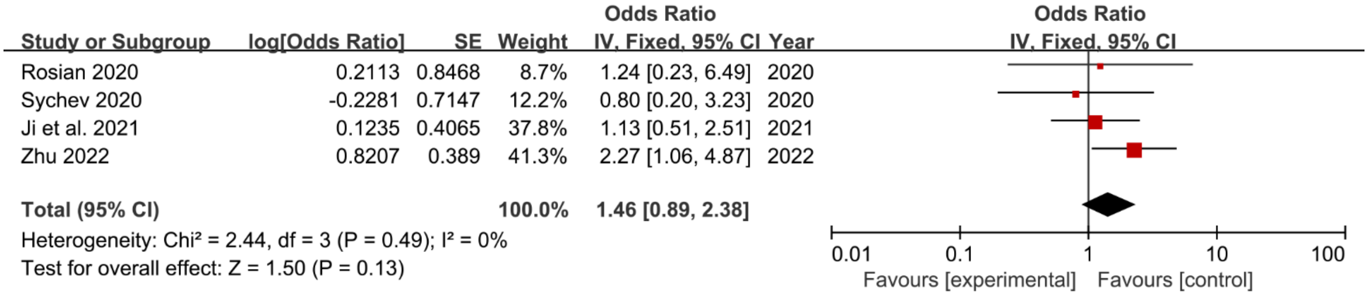

Supplementary Figure S3. Forest plots illustrating the relationship between *ABCB1* rs1045642 and bleeding rate of dabigatran. T carriers (TT + CT) versus CC carriers.

\

Study or Subgroup	Experimental Mean	Experimental SD	Experimental Total	Control Mean	Control SD	Control Total	Weight	Mean Difference IV, Random, 95% CI
**4.1.1 AA vs GG**								
Abdrakhmanov 2024	54.88	43.13	54	60.54	37.83	32	28.7%	-5.66 [-23.10, 11.78]
Cumitini 2024	72.15	28.48	17	84.2	42.95	22	22.0%	-12.05 [-34.53, 10.43]
Olšerová 2024	88.71	58.38	139	110.96	72.81	76	26.4%	-22.25 [-41.28, -3.22]
Zhu 2022	58.52	34.93	25	46.2	31.1	13	22.9%	12.32 [-9.44, 34.08]
**Subtotal (95% CI)**			**235**			**143**	**100.0%**	**-7.33 [-21.14, 6.47]**
Heterogeneity: Tau² = 93.59; Chi² = 5.70, df = 3 (P = 0.13); I² = 47%								
Test for overall effect: Z = 1.04 (P = 0.30)								
**4.1.2 GA vs GG**								
Abdrakhmanov 2024	53.72	33.71	64	60.54	37.83	32	29.3%	-6.82 [-22.31, 8.67]
Cumitini 2024	56.59	33.67	61	84.2	42.95	22	23.3%	-27.61 [-47.45, -7.77]
Olšerová 2024	87.14	59.63	217	110.96	72.81	76	25.5%	-23.82 [-42.01, -5.63]
Zhu 2022	48.8	42.93	45	46.2	31.1	13	21.9%	2.60 [-18.45, 23.65]
**Subtotal (95% CI)**			**387**			**143**	**100.0%**	**-13.94 [-27.16, -0.71]**
Heterogeneity: Tau² = 92.71; Chi² = 6.14, df = 3 (P = 0.11); I² = 51%								
Test for overall effect: Z = 2.07 (P = 0.04)								
**4.1.3 AA+ GA vs GG**								
Abdrakhmanov 2024	54.25	38.14	118	60.54	37.83	32	28.7%	-6.29 [-21.09, 8.51]
Cumitini 2024	59.98	33.07	78	84.2	42.95	22	23.0%	-24.22 [-43.61, -4.83]
Olšerová 2024	87.75	59.07	356	110.96	72.81	76	25.3%	-23.21 [-40.69, -5.73]
Zhu 2022	52.27	40.27	70	46.2	31.1	13	23.0%	6.07 [-13.29, 25.43]
**Subtotal (95% CI)**			**622**			**143**	**100.0%**	**-11.84 [-25.33, 1.64]**
Heterogeneity: Tau² = 107.89; Chi² = 7.02, df = 3 (P = 0.07); I² = 57%								
Test for overall effect: Z = 1.72 (P = 0.09)								
**4.1.4 AA vs GG+GA**								
Abdrakhmanov 2024	54.88	43.13	54	55.97	35.07	96	28.1%	-1.09 [-14.56, 12.38]
Cumitini 2024	72.15	28.48	17	63.91	38.11	83	20.3%	8.24 [-7.59, 24.07]
Olšerová 2024	88.71	58.38	139	93.32	64.25	293	34.3%	-4.61 [-16.79, 7.57]
Zhu 2022	58.52	34.93	25	48.22	40.34	58	17.3%	10.30 [-6.88, 27.48]
**Subtotal (95% CI)**			**235**			**530**	**100.0%**	**1.56 [-5.57, 8.70]**
Heterogeneity: Tau² = 0.00; Chi² = 2.81, df = 3 (P = 0.42); I² = 0%								
Test for overall effect: Z = 0.43 (P = 0.67)								
Test for subgroup differences: Chi² = 6.02, df = 3 (P = 0.11), I² = 50.2%								

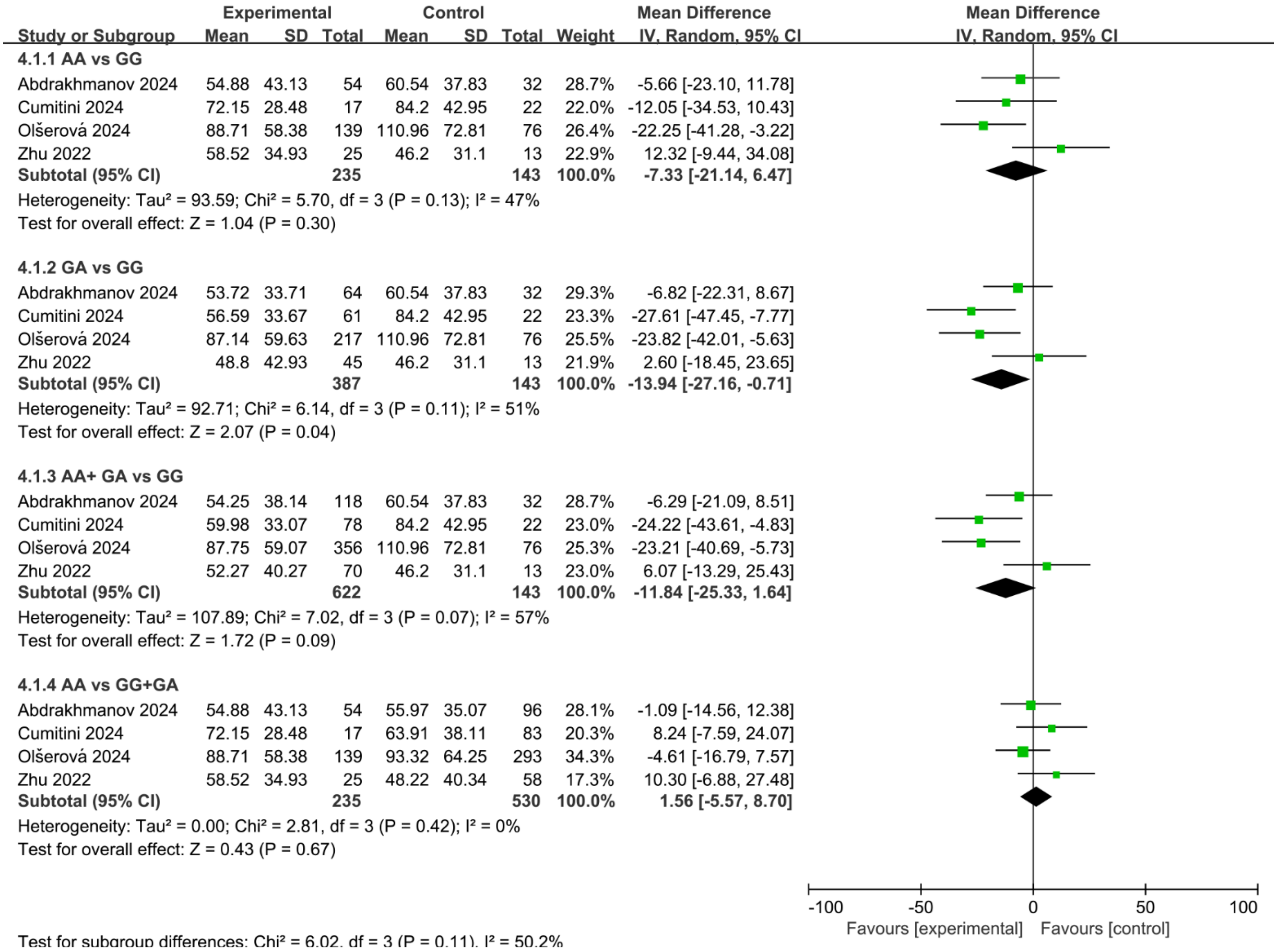

Supplementary Figure S4. Forest plots illustrating the relationship between *ABCB1* rs4148748 and C_{trough} of dabigatran.

Study or Subgroup	Experimental Mean	Experimental SD	Experimental Total	Control Mean	Control SD	Control Total	Weight	Mean Difference IV, Random, 95% CI
**4.2.1 AA vs GG**								
Abdrakhmanov 2024	108.05	77.67	54	109.18	74.98	32	28.9%	-1.13 [-34.36, 32.10]
Cumitini 2024	96.39	36.82	17	151.81	73.48	22	28.2%	-55.42 [-90.76, -20.08]
Sychev 2018	165.89	91.93	15	224.71	133.48	20	16.8%	-58.82 [-133.56, 15.92]
Zhu 2022	114.16	50.35	22	82.3	58.66	10	26.1%	31.86 [-10.15, 73.87]
**Subtotal (95% CI)**			**108**			**84**	**100.0%**	**-17.53 [-59.97, 24.92]**
Heterogeneity: Tau² = 1336.95; Chi² = 11.79, df = 3 (P = 0.008); I² = 75%								
Test for overall effect: Z = 0.81 (P = 0.42)								
**4.2.2 GA vs GG**								
Abdrakhmanov 2024	99.55	68.41	64	109.18	74.98	32	40.0%	-9.63 [-40.55, 21.29]
Cumitini 2024	131.04	90.26	61	151.81	73.48	22	28.4%	-20.77 [-58.93, 17.39]
Sychev 2018	162.87	115.48	25	224.71	133.48	20	8.6%	-61.84 [-135.81, 12.13]
Zhu 2022	97.33	60.65	26	82.3	58.66	10	23.0%	15.03 [-28.16, 58.22]
**Subtotal (95% CI)**			**176**			**84**	**100.0%**	**-11.60 [-33.82, 10.61]**
Heterogeneity: Tau² = 72.56; Chi² = 3.47, df = 3 (P = 0.32); I² = 14%								
Test for overall effect: Z = 1.02 (P = 0.31)								
**4.2.3 AA+GA vs GG**								
Abdrakhmanov 2024	103.37	72.52	118	109.18	74.98	32	33.7%	-5.81 [-34.90, 23.28]
Cumitini 2024	123.49	82.69	78	151.81	73.48	22	28.3%	-28.32 [-64.09, 7.45]
Sychev 2018	164.38	103.31	40	224.71	133.48	20	13.1%	-60.33 [-127.02, 6.36]
Zhu 2022	105.04	56.22	36	82.3	58.66	10	24.9%	22.74 [-17.99, 63.47]
**Subtotal (95% CI)**			**272**			**84**	**100.0%**	**-12.22 [-39.94, 15.51]**
Heterogeneity: Tau² = 373.01; Chi² = 5.76, df = 3 (P = 0.12); I² = 48%								
Test for overall effect: Z = 0.86 (P = 0.39)								
**4.2.4 AA vs GA+GG**								
Abdrakhmanov 2024	108.05	77.67	54	102.72	70.4	96	28.9%	5.33 [-19.72, 30.38]
Cumitini 2024	96.39	36.82	17	136.55	86.19	83	28.7%	-40.16 [-65.66, -14.66]
Sychev 2018	165.89	91.93	15	190.35	126.23	45	15.2%	-24.46 [-83.83, 34.91]
Zhu 2022	114.16	50.35	22	93.16	59.66	36	27.2%	21.00 [-7.68, 49.68]
**Subtotal (95% CI)**			**108**			**260**	**100.0%**	**-7.98 [-38.52, 22.55]**
Heterogeneity: Tau² = 677.22; Chi² = 11.42, df = 3 (P = 0.010); I² = 74%								
Test for overall effect: Z = 0.51 (P = 0.61)								

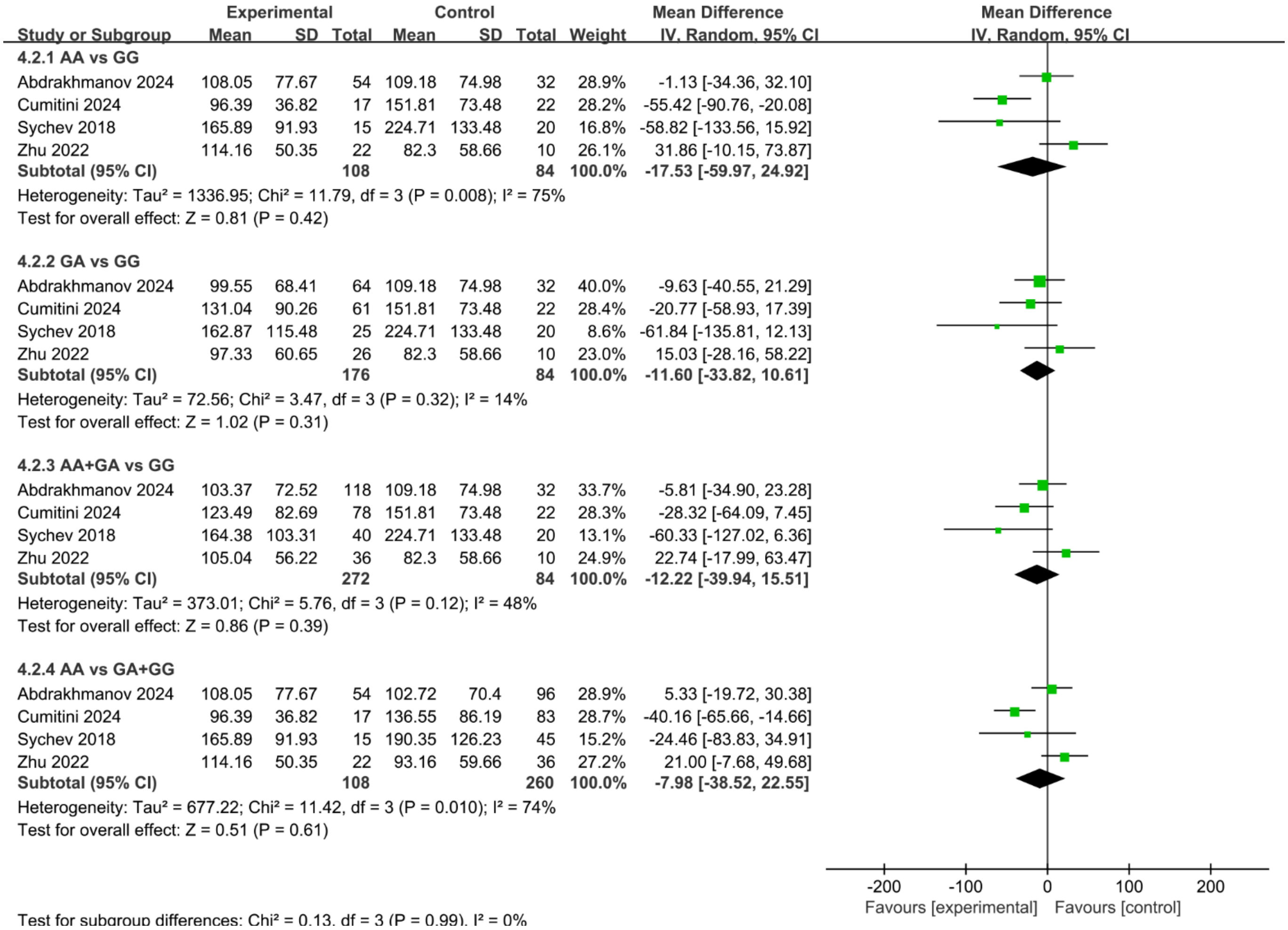

Test for subgroup differences: Chi² = 0.13, df = 3 (P = 0.99), I² = 0%

Supplementary Figure S5. Forest plots illustrating the relationship between *ABCB1* rs4148738 and C_{peak} of dabigatran.

Study or Subgroup	log[Odds Ratio]	SE	Weight	Odds Ratio IV, Fixed, 95% CI	Year
Sychev 2020	0.1823	0.835	9.5%	1.20 [0.23, 6.16]	2020
Rosian 2020	-0.8473	0.8729	8.7%	0.43 [0.08, 2.37]	2020
Ji et al. 2021	0.0796	0.4143	38.7%	1.08 [0.48, 2.44]	2021
Zhu 2022	-0.8316	0.3931	43.0%	0.44 [0.20, 0.94]	2022
**Total (95% CI)**			**100.0%**	**0.68 [0.41, 1.13]**	

Heterogeneity: Chi² = 3.29, df = 3 (P = 0.35); I² = 9%
Test for overall effect: Z = 1.49 (P = 0.14)

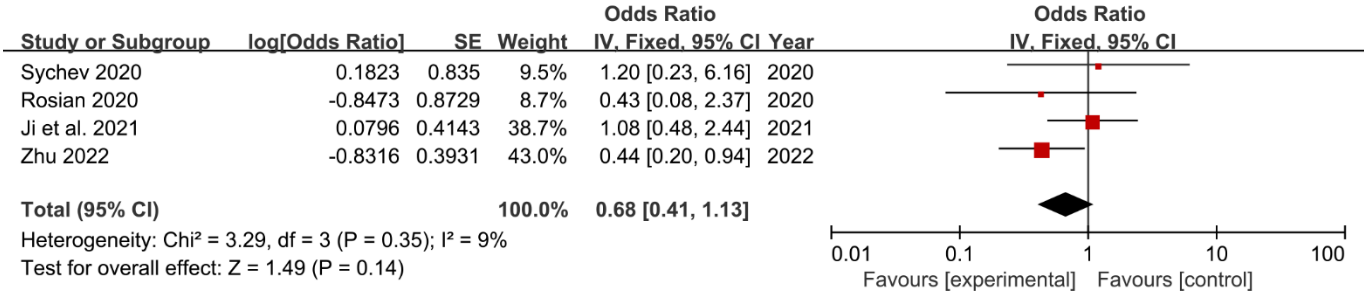

Supplementary Figure S6. Forest plots illustrating the relationship between *ABCB1* rs4148738 and bleeding rate of dabigatran. AA carriers versus G carriers (GG + GA)

Study or Subgroup	Experimental Mean	Experimental SD	Experimental Total	Control Mean	Control SD	Control Total	Weight	Mean Difference IV, Random, 95% CI
**2.1.1 AA vs GG**								
Sychev 2022	3.74	2.5	28	3.76	3.08	37	40.2%	-0.02 [-1.38, 1.34]
Wu 2023	0.73	0.69	29	1.83	2.3	15	45.4%	-1.10 [-2.29, 0.09]
Zhang 2022	5.07	5.7	38	7.94	10	74	14.4%	-2.87 [-5.78, 0.04]
**Subtotal (95% CI)**			**95**			**126**	**100.0%**	**-0.92 [-2.14, 0.30]**
Heterogeneity: Tau² = 0.49; Chi² = 3.45, df = 2 (P = 0.18); I² = 42%								
Test for overall effect: Z = 1.48 (P = 0.14)								
**2.1.2 GA vs GG**								
Sychev 2022	3.29	2.05	63	3.76	3.08	37	39.7%	-0.47 [-1.58, 0.64]
Wu 2023	1.04	1.11	51	1.83	2.3	15	38.3%	-0.79 [-1.99, 0.41]
Zhang 2022	4.11	3.92	104	7.94	10	74	22.1%	-3.83 [-6.23, -1.43]
**Subtotal (95% CI)**			**218**			**126**	**100.0%**	**-1.33 [-2.83, 0.16]**
Heterogeneity: Tau² = 1.15; Chi² = 6.31, df = 2 (P = 0.04); I² = 68%								
Test for overall effect: Z = 1.75 (P = 0.08)								
**2.1.3 AA+GA vs GG**								
Sychev 2022	3.43	2.19	91	3.76	3.08	37	40.3%	-0.33 [-1.42, 0.76]
Wu 2023	0.93	0.99	80	1.83	2.3	15	38.6%	-0.90 [-2.08, 0.28]
Zhang 2022	4.37	4.46	142	7.94	10	74	21.1%	-3.57 [-5.96, -1.18]
**Subtotal (95% CI)**			**313**			**126**	**100.0%**	**-1.23 [-2.65, 0.18]**
Heterogeneity: Tau² = 0.99; Chi² = 5.83, df = 2 (P = 0.05); I² = 66%								
Test for overall effect: Z = 1.71 (P = 0.09)								
**2.1.4 AA vs GA+GA**								
Sychev 2022	3.74	2.5	28	3.46	2.48	100	14.3%	0.28 [-0.77, 1.33]
Wu 2023	0.73	0.69	29	1.22	1.48	66	82.2%	-0.49 [-0.93, -0.05]
Zhang 2022	5.07	5.7	38	5.7	7.33	178	3.5%	-0.63 [-2.74, 1.48]
**Subtotal (95% CI)**			**95**			**344**	**100.0%**	**-0.38 [-0.78, 0.01]**
Heterogeneity: Tau² = 0.00; Chi² = 1.83, df = 2 (P = 0.40); I² = 0%								
Test for overall effect: Z = 1.91 (P = 0.06)								

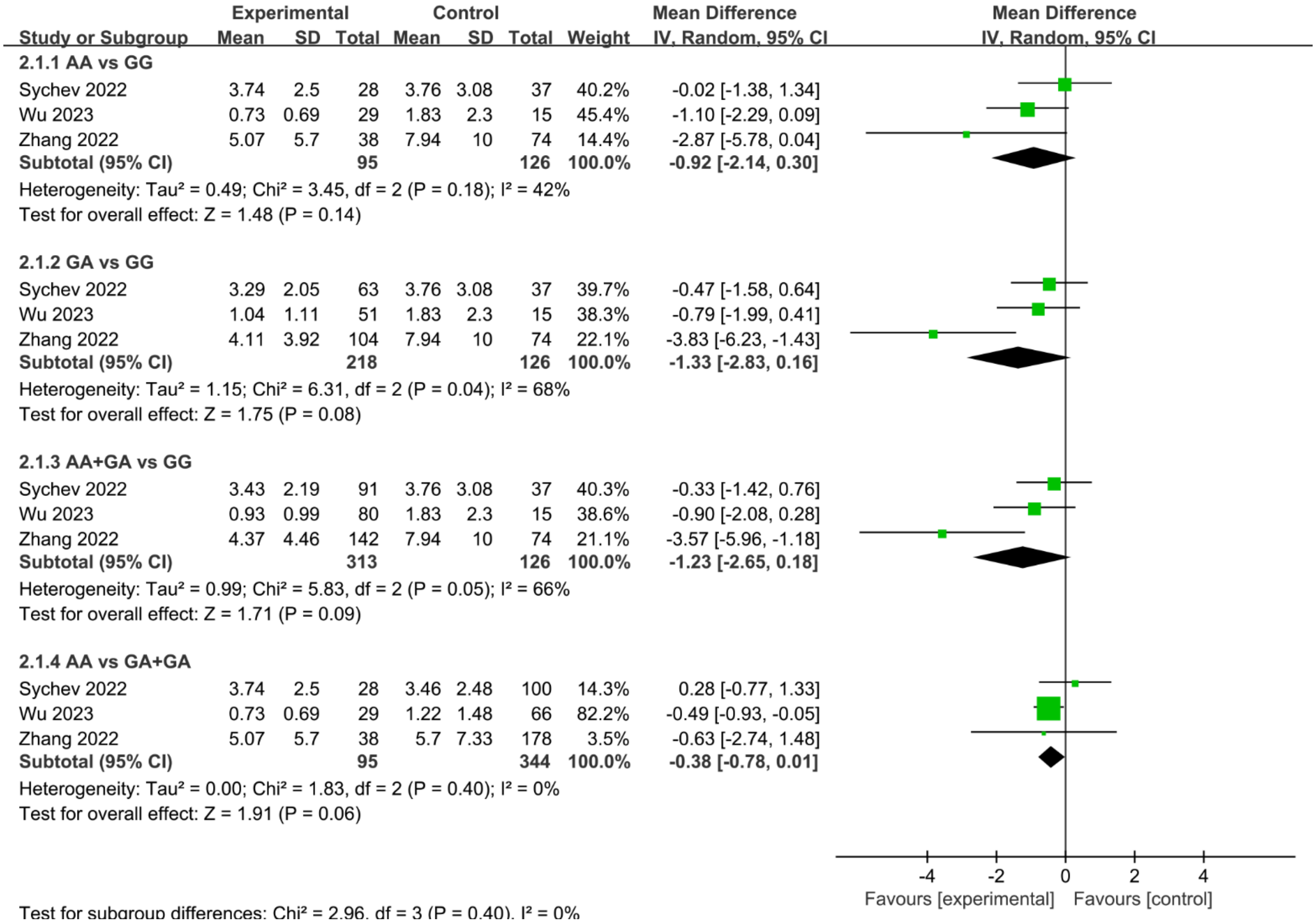

Test for subgroup differences: Chi² = 2.96, df = 3 (P = 0.40), I² = 0%

Supplementary Figure S7. Forest plots illustrating the relationship between *ABCB1* rs4148738 and C_{trough}/D of rivaroxaban.

Study or Subgroup	Experimental Events	Experimental Total	Control Events	Control Total	Weight	Odds Ratio M-H, Random, 95% CI
**2.2.1 AA vs GG**						
Ain 2024	0	9	3	28	4.2%	0.38 [0.02, 8.14]
Li 2025	17	120	8	44	21.1%	0.74 [0.30, 1.87]
Slišković 2024	17	73	17	82	24.2%	1.16 [0.54, 2.49]
Wang 2021	11	60	3	31	14.3%	2.10 [0.54, 8.15]
Wang 2025	8	27	39	66	20.4%	0.29 [0.11, 0.76]
Wu 2023	4	29	3	15	11.1%	0.64 [0.12, 3.32]
Zhang 2022	0	38	14	74	4.7%	0.05 [0.00, 0.93]
**Subtotal (95% CI)**		**356**		**340**	**100.0%**	**0.67 [0.35, 1.30]**
Total events	57		87			
Heterogeneity: Tau² = 0.32; Chi² = 10.89, df = 6 (P = 0.09); I² = 45%						
Test for overall effect: Z = 1.18 (P = 0.24)						
**2.2.2 GA vs GG**						
Ain 2024	6	29	3	28	10.3%	2.17 [0.49, 9.71]
Li 2025	41	146	39	66	17.3%	0.27 [0.15, 0.50]
Slišković 2024	26	125	17	82	16.7%	1.00 [0.51, 2.00]
Wang 2021	10	64	3	31	11.2%	1.73 [0.44, 6.79]
Wang 2025	43	103	39	66	17.2%	0.50 [0.26, 0.93]
Wu 2023	9	51	3	15	10.6%	0.86 [0.20, 3.67]
Zhang 2022	37	104	14	74	16.6%	2.37 [1.17, 4.80]
**Subtotal (95% CI)**		**622**		**362**	**100.0%**	**0.92 [0.46, 1.85]**
Total events	172		118			
Heterogeneity: Tau² = 0.62; Chi² = 26.72, df = 6 (P = 0.0002); I² = 78%						
Test for overall effect: Z = 0.22 (P = 0.82)						
**2.2.3 AA+GA vs GG**						
Ain 2024	6	38	3	28	10.4%	1.56 [0.36, 6.87]
Li 2025	58	266	39	66	17.1%	0.19 [0.11, 0.34]
Slišković 2024	43	198	17	82	16.7%	1.06 [0.56, 2.00]
Wang 2021	21	124	3	31	11.8%	1.90 [0.53, 6.84]
Wang 2025	51	130	39	66	16.9%	0.45 [0.24, 0.82]
Wu 2023	13	80	3	15	11.0%	0.78 [0.19, 3.14]
Zhang 2022	37	142	14	74	16.2%	1.51 [0.76, 3.02]
**Subtotal (95% CI)**		**978**		**362**	**100.0%**	**0.78 [0.39, 1.59]**
Total events	229		118			
Heterogeneity: Tau² = 0.68; Chi² = 30.94, df = 6 (P < 0.0001); I² = 81%						
Test for overall effect: Z = 0.68 (P = 0.50)						
**2.2.4 AA vs GA+GG**						
Ain 2024	0	9	9	57	4.8%	0.27 [0.01, 5.02]
Li 2025	17	120	80	212	20.7%	0.27 [0.15, 0.49]
Slišković 2024	17	73	43	207	20.1%	1.16 [0.61, 2.19]
Wang 2021	11	60	13	95	17.6%	1.42 [0.59, 3.41]
Wang 2025	8	27	82	169	17.6%	0.45 [0.19, 1.08]
Wu 2023	4	29	12	66	14.1%	0.72 [0.21, 2.46]
Zhang 2022	0	38	51	178	5.1%	0.03 [0.00, 0.53]
**Subtotal (95% CI)**		**356**		**984**	**100.0%**	**0.55 [0.27, 1.12]**
Total events	57		290			
Heterogeneity: Tau² = 0.56; Chi² = 20.65, df = 6 (P = 0.002); I² = 71%						
Test for overall effect: Z = 1.65 (P = 0.10)						
Test for subgroup differences: Chi² = 1.17, df = 3 (P = 0.76), I² = 0%						

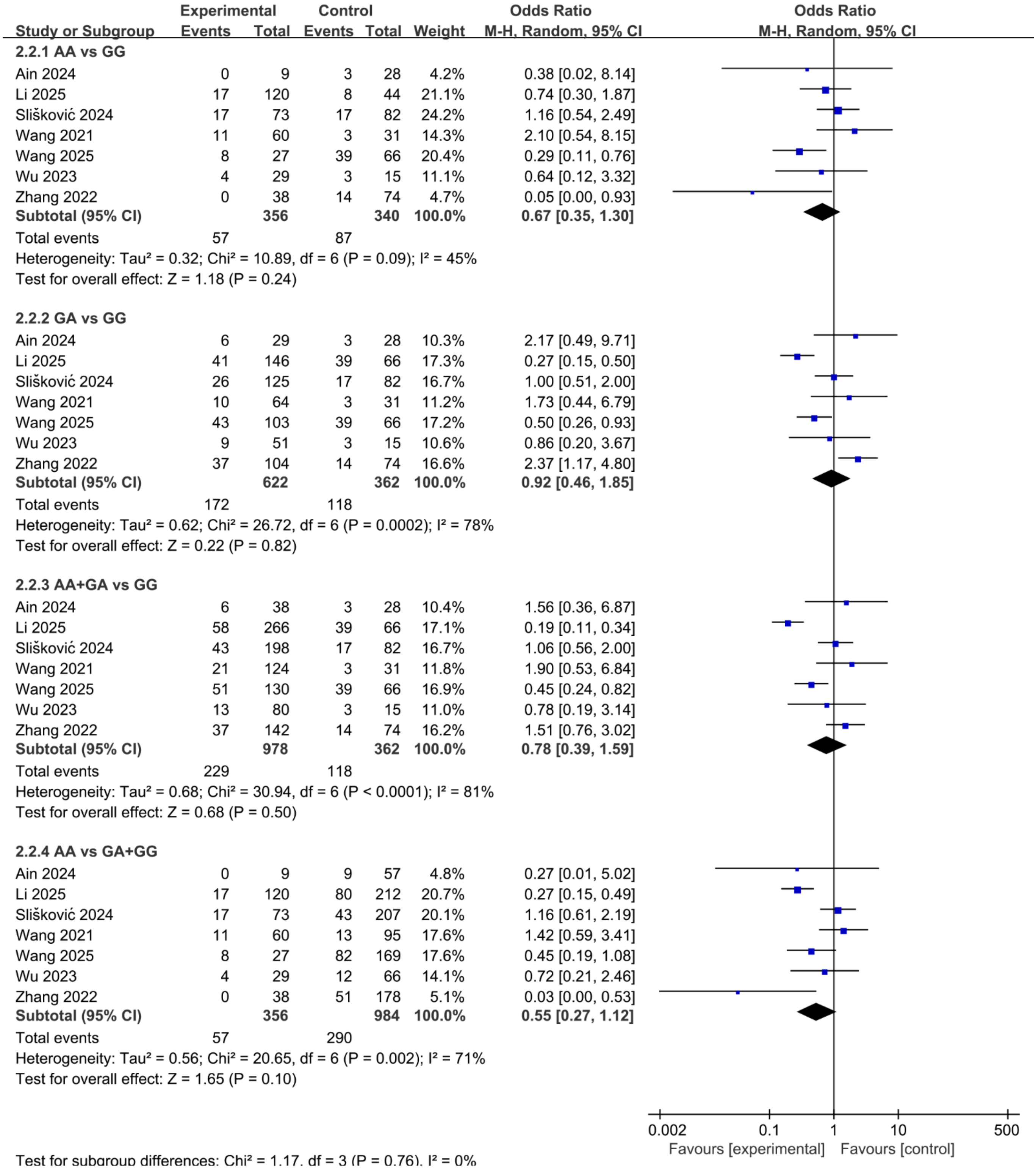

Supplementary Figure S8. Forest plots illustrating the relationship between *ABCB1* rs4148738 and bleeding rate of rivaroxaban.

Study or Subgroup	Experimental Events	Experimental Total	Control Events	Control Total	Weight	Odds Ratio M-H, Fixed, 95% CI
**3.1.1 TT vs CC**						
Ain 2024	3	25	0	6	1.6%	2.02 [0.09, 44.40]
Li 2025	26	127	7	47	19.1%	1.47 [0.59, 3.66]
Slišković 2024	11	60	21	91	32.1%	0.75 [0.33, 1.69]
Wang 2021	9	65	5	27	14.3%	0.71 [0.21, 2.35]
Wang 2025	27	72	13	21	29.6%	0.37 [0.14, 1.01]
Wu 2023	4	35	1	10	3.2%	1.16 [0.11, 11.74]
**Subtotal (95% CI)**		**384**		**202**	**100.0%**	**0.80 [0.51, 1.25]**
Total events	80		47			
Heterogeneity: Chi² = 4.52, df = 5 (P = 0.48); I² = 0%						
Test for overall effect: Z = 0.97 (P = 0.33)						
**3.1.2 CT vs CC**						
Ain 2024	6	35	0	6	1.5%	2.86 [0.14, 57.49]
Li 2025	33	136	7	47	17.1%	1.83 [0.75, 4.47]
Slišković 2024	28	129	21	91	41.8%	0.92 [0.49, 1.76]
Wang 2021	10	63	5	27	12.8%	0.83 [0.25, 2.71]
Wang 2025	51	105	13	21	24.1%	0.58 [0.22, 1.52]
Wu 2023	11	50	1	10	2.8%	2.54 [0.29, 22.27]
**Subtotal (95% CI)**		**518**		**202**	**100.0%**	**1.06 [0.71, 1.58]**
Total events	139		47			
Heterogeneity: Chi² = 4.32, df = 5 (P = 0.50); I² = 0%						
Test for overall effect: Z = 0.28 (P = 0.78)						
**3.1.3 TT+CT vs CC**						
Ain 2024	9	60	0	6	1.4%	2.40 [0.12, 46.21]
Li 2025	59	263	7	47	17.1%	1.65 [0.70, 3.88]
Slišković 2024	39	189	21	91	41.7%	0.87 [0.47, 1.58]
Wang 2021	19	128	5	27	13.0%	0.77 [0.26, 2.27]
Wang 2025	78	177	13	21	24.1%	0.48 [0.19, 1.23]
Wu 2023	15	85	1	10	2.7%	1.93 [0.23, 16.39]
**Subtotal (95% CI)**		**902**		**202**	**100.0%**	**0.95 [0.65, 1.39]**
Total events	219		47			
Heterogeneity: Chi² = 4.66, df = 5 (P = 0.46); I² = 0%						
Test for overall effect: Z = 0.28 (P = 0.78)						
**3.1.4 TT vs CC+CT**						
Ain 2024	3	25	6	41	4.2%	0.80 [0.18, 3.51]
Li 2025	26	127	40	183	27.4%	0.92 [0.53, 1.60]
Slišković 2024	11	60	49	220	18.1%	0.78 [0.38, 1.62]
Wang 2021	9	65	15	90	11.4%	0.80 [0.33, 1.97]
Wang 2025	27	72	64	126	30.6%	0.58 [0.32, 1.05]
Wu 2023	4	35	12	60	8.2%	0.52 [0.15, 1.75]
**Subtotal (95% CI)**		**384**		**720**	**100.0%**	**0.74 [0.54, 1.01]**
Total events	80		186			
Heterogeneity: Chi² = 1.63, df = 5 (P = 0.90); I² = 0%						
Test for overall effect: Z = 1.90 (P = 0.06)						

Test for subgroup differences: Chi² = 2.26, df = 3 (P = 0.52), I² = 0%

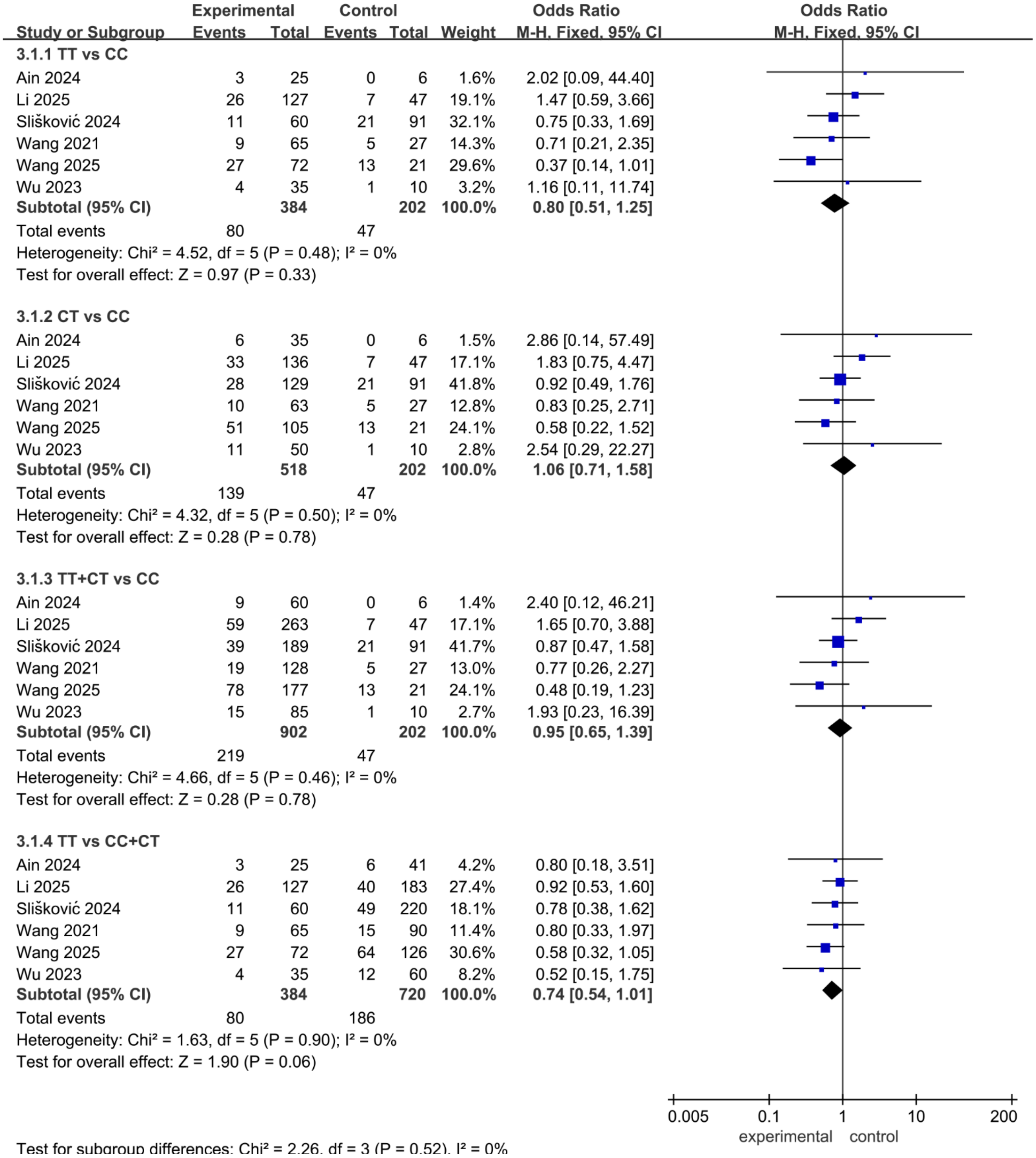

Supplementary Figure S9. Forest plots illustrating the relationship between *ABCB1* rs1128503 and bleeding rate of rivaroxaban.

Study or Subgroup	Experimental Events	Experimental Total	Control Events	Control Total	Weight	Odds Ratio M-H, Random, 95% CI
**4.1.1 AA+AT+TT vs CC**						
Ain 2024	3	28	0	9	11.3%	2.61 [0.12, 55.36]
Slišković 2024	12	59	20	86	30.0%	0.84 [0.38, 1.89]
Wang 2025	22	49	37	67	30.6%	0.66 [0.31, 1.39]
Zhang 2022	15	102	18	26	28.1%	0.08 [0.03, 0.21]
**Subtotal (95% CI)**		**238**		**188**	**100.0%**	**0.45 [0.13, 1.59]**
Total events	52		75			
Heterogeneity: Tau² = 1.20; Chi² = 16.74, df = 3 (P = 0.0008); I² = 82%						
Test for overall effect: Z = 1.24 (P = 0.22)						
**4.1.2 CT+CA vs CC**						
Ain 2024	6	29	0	9	9.0%	5.26 [0.27, 102.80]
Slišković 2024	28	135	20	86	31.9%	0.86 [0.45, 1.66]
Wang 2025	31	77	37	67	31.7%	0.55 [0.28, 1.06]
Zhang 2022	18	88	18	26	27.4%	0.11 [0.04, 0.30]
**Subtotal (95% CI)**		**329**		**188**	**100.0%**	**0.50 [0.18, 1.41]**
Total events	83		75			
Heterogeneity: Tau² = 0.76; Chi² = 13.87, df = 3 (P = 0.003); I² = 78%						
Test for overall effect: Z = 1.30 (P = 0.19)						
**4.1.3 CA+CT+AA+AT+TT vs CC**						
Ain 2024	9	57	0	9	9.9%	3.72 [0.20, 69.53]
Slišković 2024	40	194	20	86	31.1%	0.86 [0.47, 1.58]
Wang 2025	53	126	37	67	31.2%	0.59 [0.32, 1.07]
Zhang 2022	33	190	18	26	27.8%	0.09 [0.04, 0.23]
**Subtotal (95% CI)**		**567**		**188**	**100.0%**	**0.48 [0.16, 1.42]**
Total events	135		75			
Heterogeneity: Tau² = 0.90; Chi² = 18.07, df = 3 (P = 0.0004); I² = 83%						
Test for overall effect: Z = 1.33 (P = 0.18)						
**4.1.4 AA+AT+TT vs CA+CT+CC**						
Ain 2024	3	28	6	37	9.3%	0.62 [0.14, 2.73]
Slišković 2024	12	59	48	221	28.6%	0.92 [0.45, 1.87]
Wang 2025	22	49	68	144	31.7%	0.91 [0.47, 1.75]
Zhang 2022	15	102	36	114	30.4%	0.37 [0.19, 0.73]
**Subtotal (95% CI)**		**238**		**516**	**100.0%**	**0.67 [0.42, 1.09]**
Total events	52		158			
Heterogeneity: Tau² = 0.08; Chi² = 4.51, df = 3 (P = 0.21); I² = 33%						
Test for overall effect: Z = 1.62 (P = 0.11)						

Test for subgroup differences: Chi² = 0.69, df = 3 (P = 0.88), I² = 0%

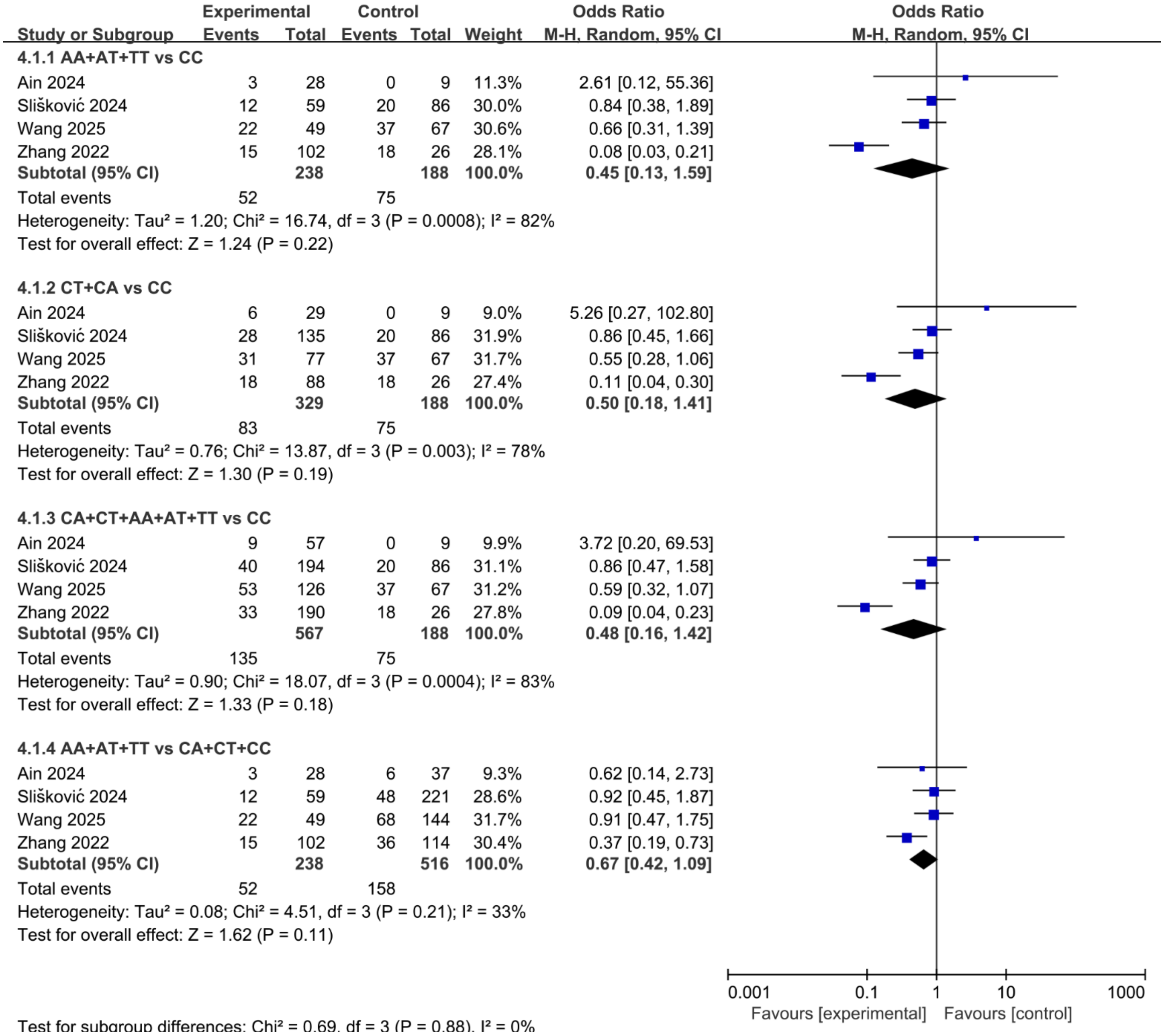

Supplementary Figure S10. Forest plots illustrating the relationship between *ABCB1* rs2032582 and bleeding rate of rivaroxaban.

Study or Subgroup	Experimental Events	Experimental Total	Control Events	Control Total	Weight	Odds Ratio M-H, Fixed, 95% CI	Year
**5.1.1 AA vs CC**							
Wu 2023	2	9	8	45	17.6%	1.32 [0.23, 7.58]	2023
Slišković 2024	1	2	49	231	3.6%	3.71 [0.23, 60.45]	2024
Li 2025	3	28	32	144	78.9%	0.42 [0.12, 1.48]	2025
**Subtotal (95% CI)**		**39**		**420**	**100.0%**	**0.70 [0.28, 1.75]**	
Total events	6		89				
Heterogeneity: Chi² = 2.52, df = 2 (P = 0.28); I² = 21%							
Test for overall effect: Z = 0.77 (P = 0.44)							
**5.1.2 CA vs CC**							
Wu 2023	6	37	8	45	13.9%	0.90 [0.28, 2.86]	2023
Slišković 2024	10	47	49	231	30.1%	1.00 [0.47, 2.16]	2024
Li 2025	31	138	32	144	56.0%	1.01 [0.58, 1.78]	2025
**Subtotal (95% CI)**		**222**		**420**	**100.0%**	**0.99 [0.65, 1.52]**	
Total events	47		89				
Heterogeneity: Chi² = 0.04, df = 2 (P = 0.98); I² = 0%							
Test for overall effect: Z = 0.03 (P = 0.98)							
**5.1.3 AA+CA vs CC**							
Wu 2023	8	46	8	45	14.1%	0.97 [0.33, 2.87]	2023
Slišković 2024	11	49	49	231	28.2%	1.08 [0.51, 2.26]	2024
Li 2025	34	166	32	144	57.7%	0.90 [0.52, 1.55]	2025
**Subtotal (95% CI)**		**261**		**420**	**100.0%**	**0.96 [0.64, 1.44]**	
Total events	53		89				
Heterogeneity: Chi² = 0.14, df = 2 (P = 0.93); I² = 0%							
Test for overall effect: Z = 0.19 (P = 0.85)							
**5.1.4 AA vs CC+CA**							
Wu 2023	2	9	14	82	16.9%	1.39 [0.26, 7.40]	2023
Slišković 2024	1	2	59	278	3.3%	3.71 [0.23, 60.23]	2024
Li 2025	3	28	63	282	79.8%	0.42 [0.12, 1.43]	2025
**Subtotal (95% CI)**		**39**		**642**	**100.0%**	**0.69 [0.28, 1.69]**	
Total events	6		136				
Heterogeneity: Chi² = 2.71, df = 2 (P = 0.26); I² = 26%							
Test for overall effect: Z = 0.81 (P = 0.42)							

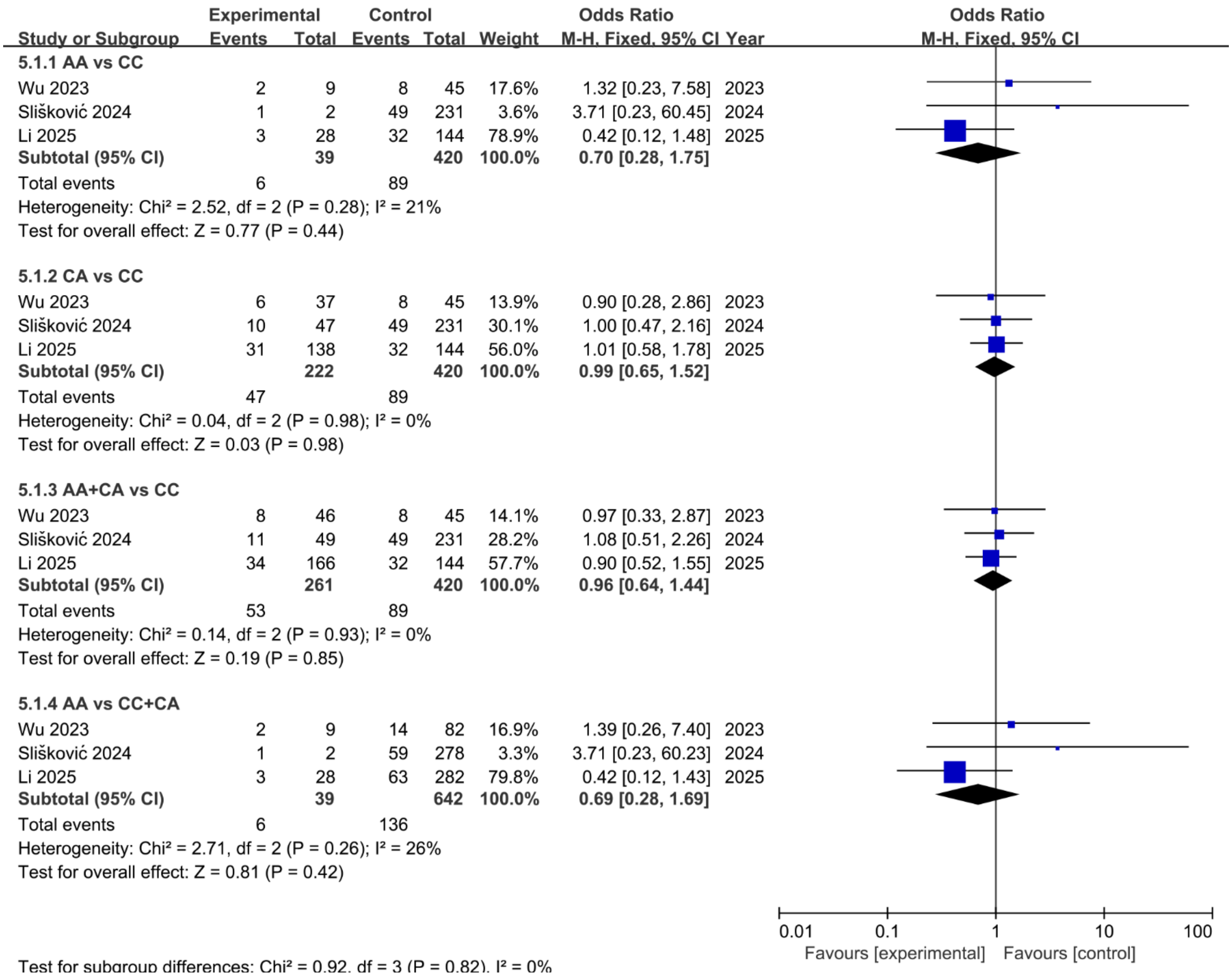

Test for subgroup differences: Chi² = 0.92, df = 3 (P = 0.82), I² = 0%

Supplementary Figure S11. Forest plots illustrating the relationship between *ABCG2* rs2231142 and bleeding rate of rivaroxaban.

Study or Subgroup	Experimental Events	Experimental Total	Control Events	Control Total	Weight	Odds Ratio M-H, Fixed, 95% CI
**6.1.1 GG vs AA**						
Ain 2024	6	39	0	3	3.2%	1.36 [0.06, 29.55]
Li 2024	9	96	0	14	3.3%	3.15 [0.17, 57.09]
Li 2025	31	155	9	33	50.5%	0.67 [0.28, 1.58]
Slišković 2024	52	245	0	2	3.3%	1.36 [0.06, 28.69]
Wu 2023	6	41	1	7	6.2%	1.03 [0.10, 10.13]
Zhang 2022	28	113	6	17	33.4%	0.60 [0.20, 1.78]
**Subtotal (95% CI)**		**689**		**76**	**100.0%**	**0.80 [0.44, 1.44]**
Total events	132		16			
Heterogeneity: Chi² = 1.56, df = 5 (P = 0.91); I² = 0%						
Test for overall effect: Z = 0.76 (P = 0.45)						
**6.1.2 GA vs AA**						
Ain 2024	3	24	0	3	3.3%	1.14 [0.05, 27.16]
Li 2024	7	55	0	14	3.0%	4.48 [0.24, 83.33]
Li 2025	26	122	9	33	49.2%	0.72 [0.30, 1.74]
Slišković 2024	8	33	0	2	3.0%	1.67 [0.07, 38.28]
Wu 2023	8	40	1	7	6.0%	1.50 [0.16, 14.29]
Zhang 2022	17	86	6	17	35.5%	0.45 [0.15, 1.39]
**Subtotal (95% CI)**		**360**		**76**	**100.0%**	**0.83 [0.45, 1.51]**
Total events	69		16			
Heterogeneity: Chi² = 2.99, df = 5 (P = 0.70); I² = 0%						
Test for overall effect: Z = 0.61 (P = 0.54)						
**6.1.3 GG+GA vs AA**						
Ain 2024	9	63	0	3	3.2%	1.22 [0.06, 25.57]
Li 2024	16	151	0	14	3.2%	3.53 [0.20, 61.98]
Li 2025	57	277	9	33	50.6%	0.69 [0.30, 1.57]
Slišković 2024	60	278	0	2	3.1%	1.38 [0.07, 29.22]
Wu 2023	14	81	1	7	6.0%	1.25 [0.14, 11.25]
Zhang 2022	45	199	6	17	33.9%	0.54 [0.19, 1.53]
**Subtotal (95% CI)**		**1049**		**76**	**100.0%**	**0.80 [0.45, 1.42]**
Total events	201		16			
Heterogeneity: Chi² = 2.08, df = 5 (P = 0.84); I² = 0%						
Test for overall effect: Z = 0.76 (P = 0.45)						
**6.1.4 GG vs GA+AA**						
Ain 2024	6	39	3	27	4.0%	1.45 [0.33, 6.40]
Li 2024	9	96	7	69	9.9%	0.92 [0.32, 2.59]
Li 2025	31	155	35	155	37.5%	0.86 [0.50, 1.48]
Slišković 2024	52	245	8	35	14.8%	0.91 [0.39, 2.12]
Wu 2023	6	41	9	47	9.6%	0.72 [0.23, 2.24]
Zhang 2022	28	113	23	103	24.2%	1.15 [0.61, 2.15]
**Subtotal (95% CI)**		**689**		**436**	**100.0%**	**0.95 [0.69, 1.32]**
Total events	132		85			
Heterogeneity: Chi² = 1.03, df = 5 (P = 0.96); I² = 0%						
Test for overall effect: Z = 0.30 (P = 0.77)						

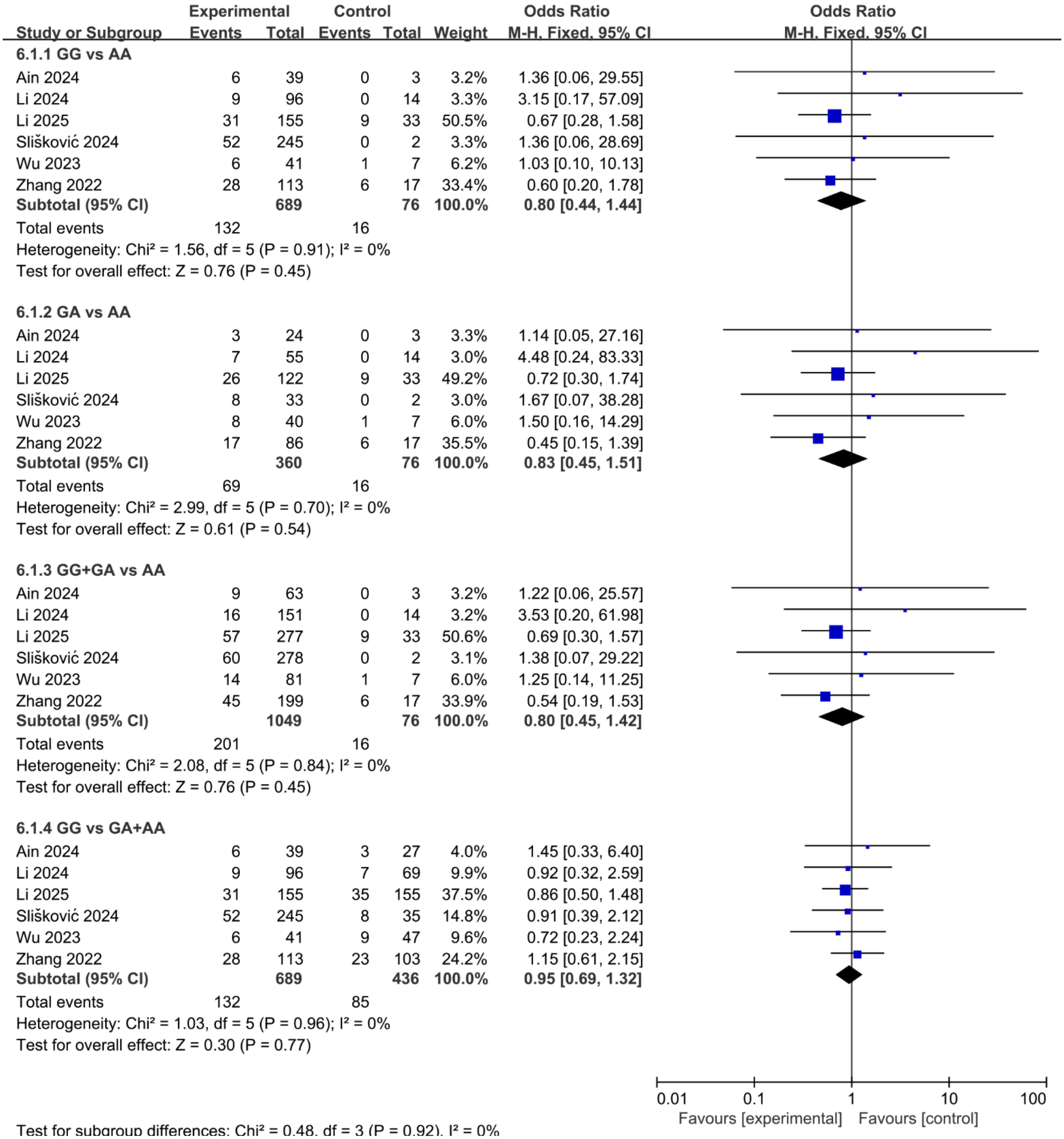

Test for subgroup differences: Chi² = 0.48, df = 3 (P = 0.92), I² = 0%

Supplementary Figure S12. Forest plots illustrating the relationship between *CYP3A5* rs776746 and bleeding rate of rivaroxaban.

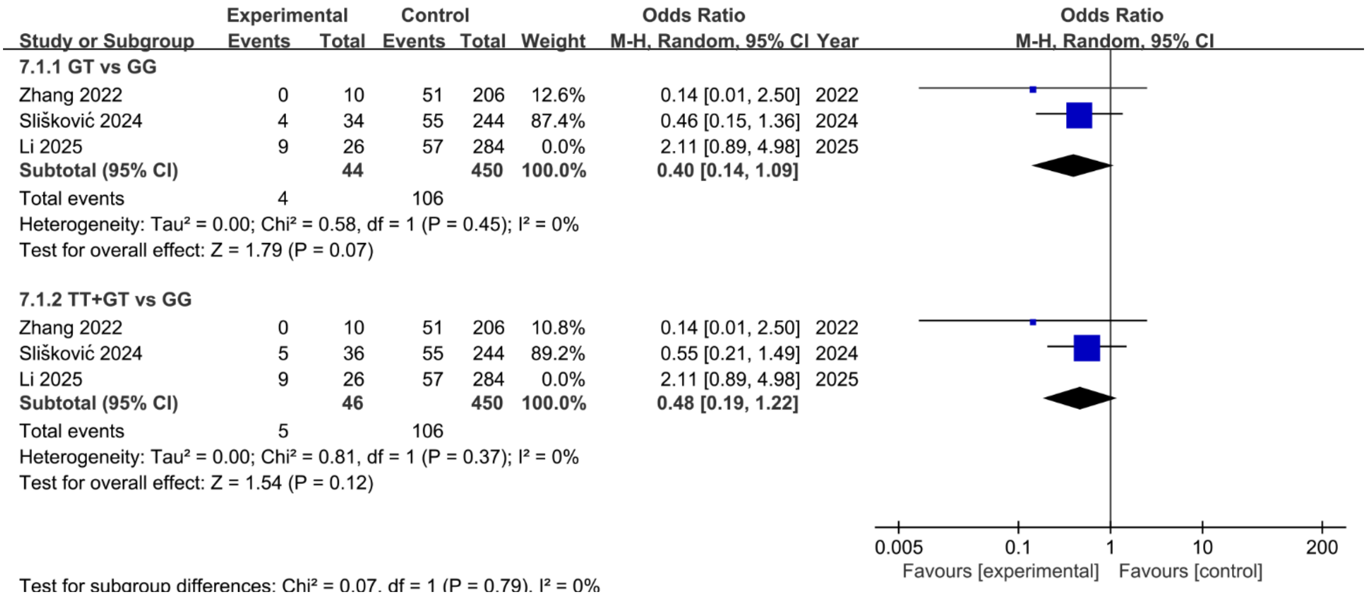

Supplementary Figure S13. Forest plots illustrating the relationship between *CYP2J2* rs890293 and bleeding rate of rivaroxaban.
